# An evaluation of inverse probability weighting using the propensity score for baseline covariate adjustment in smaller population randomised controlled trials with a continuous outcome

**DOI:** 10.1186/s12874-020-00947-7

**Published:** 2020-03-23

**Authors:** Hanaya Raad, Victoria Cornelius, Susan Chan, Elizabeth Williamson, Suzie Cro

**Affiliations:** 1 Imperial Clinical Trials Unit, Imperial College London, Stadium House, 68 Wood Lane, London, W12 7RH UK; 2 Children’s Allergy, Guy’s and St Thomas’ NHS Foundation Trust, London, United Kingdom, London, SE1 7EH UK; 3 King’s College London School of Life Course Sciences & School of Immunology & Microbial Sciences, King’s Health Partners, London, UK; 4 Department of Medical Statistics, Faculty of Epidemiology and population health, London School of Hygiene and Tropical Medicine, London, WC1E 7HT UK

**Keywords:** Randomised controlled trial, Covariate adjustment, Small population, Small sample size, Propensity score, Inverse probability weighting

## Abstract

**Background:**

It is important to estimate the treatment effect of interest accurately and precisely within the analysis of randomised controlled trials. One way to increase precision in the estimate and thus improve the power for randomised trials with continuous outcomes is through adjustment for pre-specified prognostic baseline covariates. Typically covariate adjustment is conducted using regression analysis, however recently, Inverse Probability of Treatment Weighting (IPTW) using the propensity score has been proposed as an alternative method. For a continuous outcome it has been shown that the IPTW estimator has the same large sample statistical properties as that obtained via analysis of covariance. However the performance of IPTW has not been explored for smaller population trials (< 100 participants), where precise estimation of the treatment effect has potential for greater impact than in larger samples.

**Methods:**

In this paper we explore the performance of the baseline adjusted treatment effect estimated using IPTW in smaller population trial settings. To do so we present a simulation study including a number of different trial scenarios with sample sizes ranging from 40 to 200 and adjustment for up to 6 covariates. We also re-analyse a paediatric eczema trial that includes 60 children.

**Results:**

In the simulation study the performance of the IPTW variance estimator was sub-optimal with smaller sample sizes. The coverage of 95% CI’s was marginally below 95% for sample sizes < 150 and ≥ 100. For sample sizes < 100 the coverage of 95% CI’s was always significantly below 95% for all covariate settings. The minimum coverage obtained with IPTW was 89% with *n* = 40. In comparison, regression adjustment always resulted in 95% coverage. The analysis of the eczema trial confirmed discrepancies between the IPTW and regression estimators in a real life small population setting.

**Conclusions:**

The IPTW variance estimator does not perform so well with small samples. Thus we caution against the use of IPTW in small sample settings when the sample size is less than 150 and particularly when sample size < 100.

## Background

Randomised controlled trials (RCTs) provide high quality evidence for the evaluation of new and existing treatments. The random allocation of participants to treatment groups guards against allocation bias and ensures all observed and unobserved baseline covariates are independent of treatment allocation. In expectation, participants in alternative randomized groups will differ only by their treatment allocation and any subsequent effects of that treatment. Thus any differences in outcomes between the randomised groups can be attributed to the treatment under investigation. A great deal of time, effort and money typically goes into setting up and running RCTs. It is therefore important to estimate the treatment effect accurately and with optimal precision in the analysis.

One way to increase precision in the estimate and improve power for RCTs with continuous outcomes is through adjustment of pre-specified prognostic baseline covariates [1–5]. It has been shown that the greater the correlation between a covariate and the outcome, the greater the reduction in the standard error of the treatment effect [1, 3]. Kahan [5] demonstrated substantial gains in precision when adjustments for highly prognostic covariates were made. For these reasons European Medicines Agency (EMA) guidelines [6] recommend investigators consider adjusting treatment effect estimates for variables known a-priori to be strongly related with outcome. In line with the EMA guidelines [6] we stress that the pre-specified nature of any baseline adjustment is crucial in the RCT setting. Covariates to be adjusted for must be pre-specified in the trial protocol and/or statistical analysis plan based on previous evidence and clinical knowledge. Throughout this article we assume all adjustments are pre-specified and do not consider post-hoc adjustment further. Issues associated with post-hoc adjustments, including the potential for cherry picking the most beneficial result, have been debated elsewhere [3, 7, 8].

Adjustment for any stratification variables used within the randomisation is also important to obtain correct standard errors (SE’s) and no loss of power [9, 10]. Adjustment can also be especially useful to account for any chance imbalances in prognostic baseline covariates. Although the process of randomisation ensures there is no confounding, it will not always result in a perfect balance across baseline covariates between treatment groups. As discussed by Senn [11] “there is no requirement for baseline balance for valid inference,” but where imbalance occurs the treatment effect may not be estimated so precisely (larger standard errors will be obtained).

Since in smaller trial settings there will be a greater chance of imbalance [12], pre-specified adjustment to allow for any chance imbalances in prognostic baseline covariates can be particularly useful to achieve a more precise answer. Although similar gains in efficiency will be realized in both small and large sample size settings through adjustment for any chance imbalance [11, 12]. Additionally in smaller populations settings Parmar et al. [13] discuss how the benefits of adjustment for baseline covariates could be harnessed to inform the trial design. Since adjusting for covariates which are associated with the outcome leads to increases in power, in smaller population settings a lower sample size can be justified taking into account the proposed adjustment, in comparison to that required for an unadjusted analysis. Thus appropriate statistical methods for performing adjusted analyses are important for various reasons and may be particularly useful in smaller trial settings.

Adjustment for baseline variables in the analysis of RCTs is typically done using regression methods. For example, for a continuous outcome a linear regression model may be utilized. Recently, as an alternative method of covariate adjustment, Williamson et al. [14] proposed Inverse Probability of Treatment Weighting (IPTW) using the estimated propensity score. They showed that, for a continuous outcome, the IPTW treatment estimator has the same large sample statistical properties as that obtained via Analysis of Covariance (ANCOVA).

Since baseline covariates are not included in the outcome model when IPTW is employed we hypothesized this approach might confer some advantages in small sample RCT settings where adjustment for a number of baseline covariates is required. However, the theory of Williamson et al. used to derive the properties of the IPTW treatment and variance estimator used large sample properties and simulations only explored performance down to a sample size of 100.

The aim of this paper is to explore the performance of IPTW using the propensity score and to compare it with the more commonly used linear regression baseline adjustment approach in smaller population trial settings. In the next section we outline the baseline covariate adjusted regression method and IPTW propensity score approach in more detail. In Section 3 we assess how IPTW and linear regression adjustment compare in small population trial settings using a simulation study. Since the computation of the appropriate IPTW variance estimate that accounts for the uncertainty in the estimated propensity score involves a number of computational steps (outlined in Section 2.2), we also examine the performance of the bootstrap variance for the IPTW treatment estimate. In Section 4 we re-analyse a paediatric eczema RCT involving 60 children. We finish with a discussion and recommendations in Section 5.

## Methods

### Regression modelling

Typically, adjustment for pre-specified baseline covariates in the analysis of RCTs is performed using standard regression methods. Consider a two arm randomised trial with a total of n subjects where for participant i, Z_i_ = 0 or 1 represents treatment allocation (0 = control, 1 = treatment), Y_i_ denotes the outcome of interest and ***X***_*i*_ = (*X*_*i*1_, …, *X*_*ip*_)′, a (p × 1) vector of baseline covariates. For a continuous outcome Y_i_, a linear regression model with the following structure may be used to estimate the baseline adjusted treatment effect,

$$ {Y}_i=\alpha +\theta {Z}_i+\beta {\boldsymbol{X}}_i+{e}_i $$

$$ {e}_i\sim N\left(0,{\sigma}^2\right) $$

Here θ represents the treatment effect after adjustment for **X**_i_ i.e. conditional on having particular baseline covariate values of **X**_i_. Often this is referred to as an analysis of covariance (ANCOVA). For other types of outcomes alternative models can be used, such as a logistic regression for binary outcomes or a Cox proportional hazards model for time to event outcomes [4].

Previous research has explored the properties of linear regression estimators with a varying number of subjects. Various rules-of-thumb for the number of subjects required in linear regression analyses have been debated, which include specifying either a fixed sample size, regardless to the number of predictors, or a minimum number of subjects per variable (SPV) ranging from 2 to 20 [15–17]. In this study, we will compare the performance of linear regression modelling for covariate adjustment in smaller sample RCT settings against IPTW using the propensity score.

### IPTW propensity score approach

The propensity score is defined as the conditional probability of being exposed to a particular treatment given the values of measured covariates. For example, continuing in the above two arm RCT setting where *Z* denotes treatment allocation, Y the continuous outcome and the baseline covariates are represented as ***X*** = (*X*_1_, …, *X*_*p*_), the propensity score is defined as:

$$ e(X)=\mathbb{P}\left(Z=1\ \right|\ \boldsymbol{X}\Big). $$

In a simple two arm RCT allocating individuals in a 1:1 ratio this is known to be 0.5. But, previous work has shown that estimating the propensity score using the observed data and using it as if we didn’t know the true score provides increased precision without introducing bias in large samples [14]. The most popular model of choice for estimating the propensity score is a logistic regression [18]. As the treatment indicator *Z* is binary, and suppose the logistic regression is parametrised by ***α*** = (*α*_0_, *α*_1_, …, *α*_*p*_)^⊤^, so that:

$$ \mathit{\log}\left\{\mathrm{e}\left(\boldsymbol{X}\right)\right./\left(1-\mathrm{e}\left(\boldsymbol{X}\right)\right)\Big\}={\boldsymbol{X}}^{\top}\boldsymbol{\alpha} . $$

For each participant indexed by the subscript i, a probability of being either in the treatment or control arm, given the baseline characteristics, can be estimated from the fitted propensity score model as:

$$ {\hat{e}}_i=\hat{e}\left({\boldsymbol{X}}_i\right)=\frac{\mathrm{e} xp\left({\boldsymbol{X}}_i^{\top}\hat{\boldsymbol{\alpha}}\right)}{1+\mathrm{e} xp\left({\boldsymbol{X}}_i^{\top}\hat{\boldsymbol{\alpha}}\right)} $$

As described in [18] other methods can be used to obtain the propensity score such as neural networks, recursive partitioning and boosting, however we focus on estimation via logistic model throughout.

The propensity score was originally introduced in 1983 by Rosenbaum and Rubin [19] as a tool to adjust for confounding in the observational study setting. Rosenbaum and Rubin showed that, under certain assumptions, at each value of the propensity score the difference between treatment arms will be an unbiased estimate of the treatment effect at that value. At each value of the propensity score individuals will on average have the same distribution of covariates included in the propensity score model. Consequently matching on the propensity score, stratification on the propensity score or covariate adjustment using the propensity score can provide an unbiased estimate of the treatment effect. Alternatively Inverse Probability of Treatment Weighting (IPTW) using the propensity score [20] may be used. That is for participants in a treatment arm a weight of ${w}_i=1/{\hat{e}}_i$ is assigned, while participants in a control arm are assigned weights of ${w}_i=1/\left(1-{\hat{e}}_i\right)$. For a continuous outcome, the adjusted mean treatment group difference can then be obtained by fitting a linear regression model on treatment only, weighted by the inverse probability of receiving treatment.

Unlike within the observational setting, issues of confounding do not occur in the RCT setting. However, recently Williamson et al. [14] introduced the propensity score approach, specifically via IPTW, as a useful method for covariate adjustment to obtain variance reduction in RCT settings. Crucially the variance estimator needs to take into account the estimation of the propensity score. Williamson et al. showed consistent estimation of the treatment effect and large sample equivalence with the variance estimated via ANCOVA using their derived variance estimator, which is based on the theory of M-estimation [21] and Lunceford and Davidian [20] and takes into account the estimation of the propensity score. That is, the full sandwich variance estimator, which taken into account all the estimating equations including the components estimating the propensity score. We hence forth refer to this variance estimator as the IPTW-W variance estimator (see eq. 1, Additional file 1). There has already been examples where trialists have used such methods to obtain precise estimates in the RCT setting [22].

### Simulation study

To assess the performance of the two methods of baseline covariate adjustment in small population RCT settings we conducted a simulation study. We also explored the performance of the non-parametric bootstrap variance for the IPTW treatment estimator since the IPTW-W variance estimate involves a number of computational steps (see eq. 1, Appendix A, Additional file 1). Data generation and all analyses were conducted using Stata [23].

#### Data generation

First we considered RCT scenarios with continuous covariates. We generated a set of 6 continuous covariates (C1-C6), as independent standard normal variables with mean 0 and a variance of 1. A treatment arm indicator (Z) was generated from a Bernoulli distribution with a probability of 0.5 to obtain approximately equally sized treatment groups. A normally distributed outcome Y, with mean E [Y] = 2*C1 + 2*C2 + 2*C3 + 2*C4 + 2*C5 + 2*C6 + 5*Z and variance 5^2^ was then simulated. Covariates were therefore moderately associated with the outcome with a one standard deviation increase in the variable associated with a 2 unit increase in outcome. Treatment was simulated to have a stronger association with outcome, with a true difference of θ = 5 in outcome between treatment arms. Fixed sample sizes of 40–150 (in multiples of 10) and 200 were drawn. The parameters chosen ensured all trial scenarios had at least 80% power. For each sample size scenario we randomly generated a total of 2000 datasets. Secondly we repeated the above steps but included a mix of binary and continuous covariates (see Additional file 1 for these additional simulation methods).

#### Statistical analysis

A linear regression model containing the treatment covariate only was fitted to estimate the unadjusted treatment effect for each simulated data set. Subsequently we conducted four different adjusted analyses, adjusted for (i) C1 only, (ii) C1-C2, (iii) C1-C4 and (iv) C1-C6. Adjusted analyses were performed using multiple linear regression and IPTW using the propensity score estimated via a logistic regression. The outcome model used in the IPTW analysis was a linear regression of outcome on treatment, weighted by the estimated propensity score. For each analysis we extracted the estimated treatment effect, $\hat{\theta}$, and its estimated standard error, $\hat{SE}$. For the unadjusted and adjusted linear regression analyses the model based estimated standard error was used and for IPTW we estimated the variance using the formula provided by Williamson et al. that takes into account the uncertainty in the estimated propensity score (IPTW-W¸ eq. 1 in Appendix A, Additional file 1). For each analysis we also estimated the non-parametric bootstrap variance for the treatment effect, ${\hat{SE}}_{boot}$ using 1000 replicates drawn with replacement [24] to compare with the model based and IPTW-W variance estimators. For IPTW the bootstrap included re-estimation of the propensity score for each bootstrap sample. 95% confidence intervals were calculated using the t-distribution for the linear regression analyses. For IPTW we calculated 95% confidence intervals (CI’s) using the normal distribution following the approach taken by Williamson et al.

For each scenario and analysis method we calculated the mean treatment effect and mean $\hat{SE}$ over the 2000 replicated data sets. Mean percentage bias was computed as $\Big(\hat{\theta}$–θ)/θ *100. We compared the mean estimated standard error, $\hat{SE},$ to the empirical SE of the observed treatment effect over the 2000 simulations, $SE\left(\hat{\theta}\right)$, and computed the ratio of the mean estimated SE to the empirical SE. We also calculated the coverage of the 95% CI’s as the percentage of estimated 95% CI’s that included the true value of the treatment effect (θ = 5). We used 2000 simulations for each scenario so that an empirical percentage coverage greater than 96% or less than 94% could be considered as being significantly different to the desired 95%.

### Case study: the ADAPT trial

The Atopic Dermatitis Anti-IgE Paediatric Trial (ADAPT), conducted by Chan et al. (2018), was a double-blind placebo-controlled trial of Omalizumab (anti-IgE therapy) amongst children with severe atopic eczema. A total of 62 participants were randomised to receive treatment for 24 weeks (30 omalizumab: 32 placebo) at a single specialist centre, stratified by age (< 10, ≥10 yrs) and IgE (≤1500, > 1500 IU/ml). The primary objective was to establish whether Omalizumab was superior to placebo with respect to disease severity. Outcomes of eczema severity included the total SCORing Atopic Dermatitis (SCORAD) and Eczema Area and Severity Index (EASI). Quality of life was assessed using the (Childrens) Dermatological Life Quality Index (C)DLQI. Full details of the trial protocol, statistical analysis plan and results have been published elsewhere [25–27]. Analysis followed the Intention-to-treat principle, including all individuals who received treatment as randomised with an available follow-up. Two participants were missing week 24 follow-up are not included in our analyses since the focus of these evaluations is not on missing data here.

For each outcome (the total SCORAD, EASI and (C)DLQI) a linear regression model adjusted for the baseline outcome, IgE and age was fitted. We then implemented IPTW using the propensity score, estimated via a logistic regression, including baseline outcome, IgE and age as covariates. The outcome model used in the IPTW analysis was a linear regression of the outcome on treatment, weighted by the estimated propensity score. The variance of the IPTW treatment estimate was computed using the IPTW-W variance estimator that incorporates the uncertainty in the estimated propensity score (see Eq. 1 in Additional file 1 and Stata code in Additional file 2). Following Williamson et al. we calculated 95% CI’s and *p*-values for the IPTW treatment estimate using the normal distribution. For the adjusted regression analyses we used the t-distribution. For both methods we also calculated the bootstrap variance, using 10,000 bootstrap samples drawn with replacement and included re-estimation of the propensity score for each replicate. For comparison we also performed an unadjusted analysis for each outcome, using a linear regression of outcome on treatment group only. Our focus is on evaluating the performance of IPTW against linear regression, rather than the clinical interpretation of the trial results which has been discussed elsewhere. All statistical analysis was conducted using Stata [23]. Stata code for the analysis can be found in Additional file 2.

## Results

### Results of the simulation studies

**Fig. 1:**
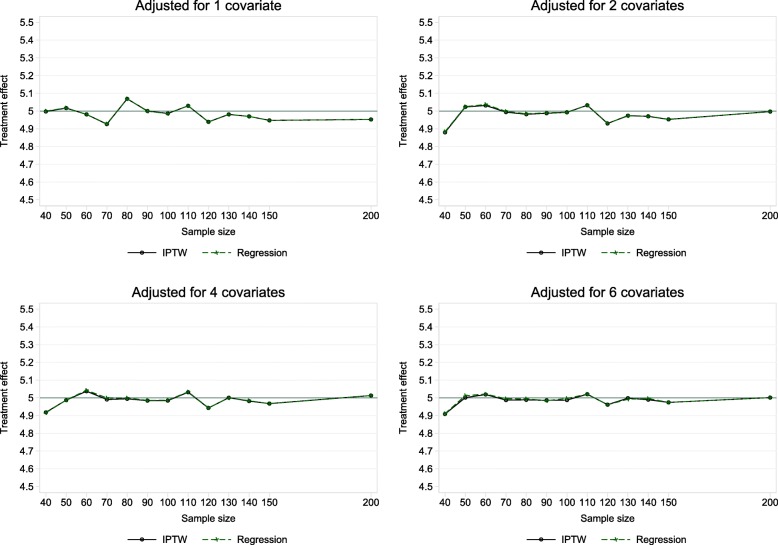
Mean treatment effect estimates in adjusted analysis performed by multiple linear regression and IPTW. True treatment effect = 5. In most cases the difference between the IPTW and regression estimate is negligible, therefore the lines are coinciding

Initially with all continuous covariates we considered 52 scenarios (13 different sample sizes and 4 levels of adjustment). The full results can be found in Supplementary Table B1, Additional file 1. Figure 1 shows the average treatment effect against sample size. There was negligible bias for the treatment effect for all settings and both methods of adjusted analysis. The treatment effects were practically equivalent when analysis was performed by regression or IPTW using the propensity score. The absolute mean percentage bias was ≤2.4% for each adjusted analysis method across all settings.

**Fig. 2:**
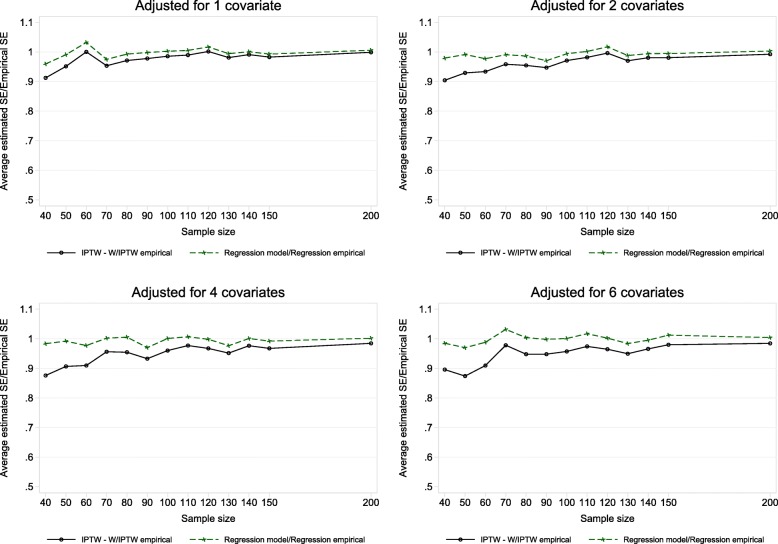
Mean estimated standard error versus empirical standard error of estimated treatment effects in adjusted analysis

Figure 2 shows how for analysis performed using linear regression, for all sample size and covariate settings the model based SE estimates were practically equivalent to the empirical standard error of the treatment effect estimates. This was the case even in the smallest sample size setting of *n* = 40 with adjustment for 6 covariates. When analysis was performed using IPTW, for sample sizes greater than 100 there were no observable differences between the estimated standard error of the IPTW estimate versus the empirical standard error for all covariate settings. With up to (and including) 6 covariates $\hat{SE}/ SE\left(\hat{\theta}\right)$ ≥0.95 and ≤ 1.00 for IPTW and *n* > 100 (see Supplementary Table B1, Additional file 1). However for smaller sample sizes (≤100) the estimated standard error of the treatment effect was smaller in comparison to the empirical standard error of the IPTW treatment estimate. This was true for all covariate settings, although less notable with adjustment for 1 covariate. The estimated standard error for the IPTW treatment estimate was typically lower than the regression standard error. When we compared the performance of the empirical standard error for the linear regression and IPTW estimate this was fairly similar when 1 to 4 covariates were adjusted for. With 6 covariates there was evident divergence between the empirical standard error for the two methods for smaller sample sizes.

**Fig. 3:**
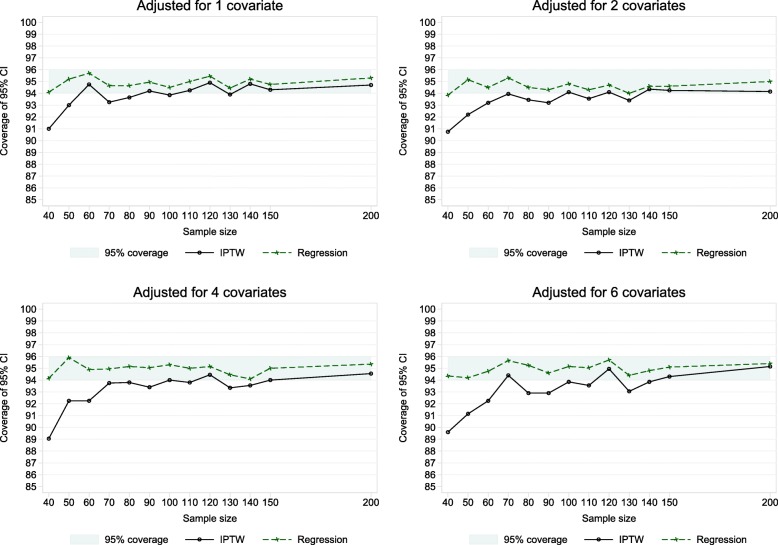
Coverage rates of 95% confidence intervals in adjusted analysis

In Fig. 3 we see the discrepancy between the IPTW-W estimate of variance and the empirical variance results in the percentage coverage being significantly less than 95% for IPTW for smaller sample sizes. The 95% coverage was first significantly below 95% (indicated by being lower than 94% with *n* = 2000 simulations) for a sample size of *n* = 130 with 1 and 2 covariates. With 4 and 6 covariates this occurred at *n* = 140. The drop in coverage was evidently only marginally significantly below 95% for sample sizes of *n* = 100–150. For sample sizes less than *n* = 100 in all covariate settings the drop in coverage becomes larger. For the smallest sample size of *n* = 40 the percentage coverage was 91.0, 90.8, 89.1 and 89.6% with adjustment for 1, 2, 4 and 6 covariates. In comparison, for the adjusted linear regression analyses the percentage coverage was not significantly different to 95% in all cases, bar marginally for *n* = 40 and 2 covariates.

#### Propensity score diagnostics

In each simulated scenario we also measured the standardised differences for the included covariates and averaged these over the 2000 simulations to assess the covariate distribution between the treatment groups. On average, as expected due to randomisation, in all scenarios (sample size 40 to 200, with 1 to 6 covariates) the average standardised differences were 0.00. For the smallest sample size of 40 and adjustment for 1, 2, 4, and 6 covariate the average non-overlap for the estimated propensity score across treatment arms was *n* = 4.3, 4.9, 6.2 and 7.6; however, over the 2000 simulations the non-overlap range was (2 to 16), (2 to 23), (2 to 27) and (2 to 29) respectively for 1, 2, 4, and 6 covariates. For a sample size of 100 the average (range) overlap with 1, 2, 4, and 6 covariates was respectively, *n* = 4.2 (2 to 17), *n* = 4.5 (2 to 18), *n* = 5.2 (2 to 19) and *n* = 6.0 (2 to 24). For a larger sample size of 200 the average (range) overlap with 1, 2, 4, and 6 covariates was respectively, *n* = 4.2 (2 to 15), *n* = 4.4 (2 to 17), *n* = 5.1 (2 to 24) and *n* = 5.4 (2 to 22).

#### Bootstrap variance

**Fig. 4:**
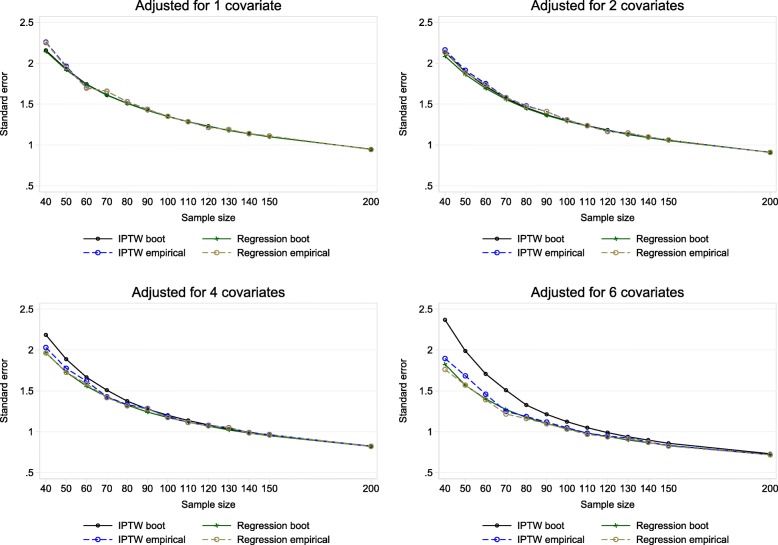
Mean bootstrap standard error versus empirical standard error of estimated treatment effects in adjusted analysis

With adjustment for 1 or 2 covariates, Fig. 4 shows that the bootstrap standard error for the IPTW treatment estimate performed similarly to the bootstrap standard error for the regression treatment estimate for all sample sizes. With 4 or more covariates performance depended on sample size and the number of covariates adjusted for. With a larger sample size of 150 and 200 the bootstrap variance for the IPTW estimate performed well in all covariate settings explored (see Supplementary Table B2, Additional file 1).

Simulation results with a mix of binary and continuous variables were generally the same and are shown in Supplementary Table B3 and B4, Additional file 1.

### Results of the case study

Table 1 summarises selected baseline characteristics, including the randomisation stratification factors, for the 60 participants in the ADAPT study included within our analyses. Generally there was a good balance in the covariate distributions between the treatment arms. Notably there were only 3 individuals with IgE ≤ 1500. We therefore also conducted sensitivity analysis treating IgE as a continuous variable (see Supplementary Table C1 in Additional file 1). The correlation between the baseline value and week 24 outcome was 0.25 for total SCORAD, 0.25 for EASI and 0.20 for (C) DLQI indicating a mild positive linear relationship between baseline and follow-up outcomes.

**Table 1:** Baseline covariates in the ADAPT study

Baseline covariate	Omalizumab *N* = 30	Placebo *N* = 30	Total *N* = 60
Age: < 10 yrs	14 (47%)	15 (50%)	29 (48%)
≥10 yrs	16 (53%)	15 (50%)	31 (52%)
Total IgE: ≤1500	1 (3%)	2 (7%)	3 (5%)
> 1500	29 (97%)	28 (93%)	57 (95%)
Total IgE (kU/l)	8110.5 (4556.0, 22,122.0)	8372 (4461.0, 16,200.0)	8321 (4508.5, 19,425.0)
Total SCORAD	69.5 (10.7)	69.5 (9.2)	69.5 (9.9)
EASI	45.5 (10.1)	43.4 (11.3)	44.4 (10.7)
(C)DLQI	17 (5.6)	17.2 (4.4)	17.1 (5.0)

Data are presented as mean (SD) for approximately normally distributed continuous values, or median (25th, 75th centile) for skewed continuous variables, and frequency (%) for categorical variables

**Fig. 5:**
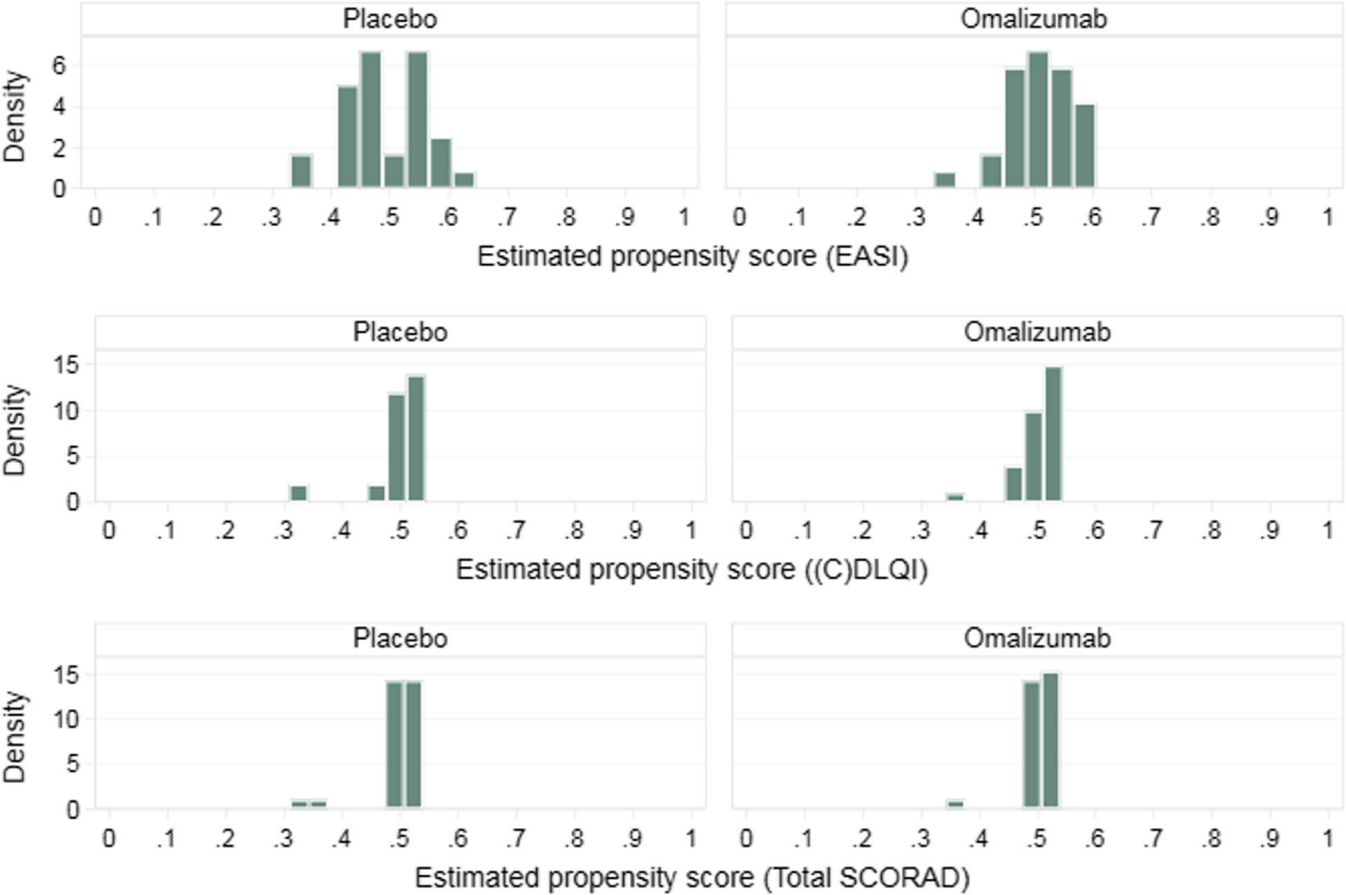
Propensity score distributions by treatment arm for the ADAPT case study

Figure 5 shows the distribution of the estimated propensity scores by treatment arm, for each outcome. Due to randomised treatment allocation, the distributions are well balanced across arms. The median (range) estimated propensity score for the placebo and omalizumab group were respectively 0.50 (0.31, 0.53) and 0.51 (0.35, 0.54) for the total SCORAD; 0.48 (0.33, 0.61) and 0.51 (0.33, 0.60) for the EASI outcome; and 0.50 (0.31, 0.54) and 0.51 (0.35, 0.54) for the (C) DLQI outcome. Despite the small sample size, due to randomization there was generally excellent overlap with non-overlap being observed for only 1 placebo and 4 omalizumab for the total SCORAD (total 8% participants), 2 placebo and 1 omalizumab for the EASI and (C) DLQI (total 5% participants).

Table 2 displays the results of the analyses where IgE was treated as a binary covariate as pre-planned in the ADAPT statistical analysis plan. For each outcome, as expected, we observed relative to the unadjusted analysis the treatment effect increased and the standard error (SE) decreased in the adjusted analyses performed by linear regression, indicating a more precise estimate. In the adjusted regression analysis for the total SCORAD the SE was 4.3% smaller, for EASI the SE was 3% smaller and for (C) DLQI the SE was 1.4% smaller in comparison to adjusted analysis. Within the IPTW analyses the obtained treatment effects did not exactly correspond with those obtained via linear regression. Moreover the standard error was consistently smaller when IPTW was used in comparison to the adjusted linear regression analysis across all 4 outcomes. For IPTW the SE for the obtained treatment effect, in comparison to the adjusted linear regression analysis was 4.5% lower (3.43 versus 3.59) for the total SCORAD, 4.6% lower for EASI (3.11 versus 3.26) and for (C) DLQI 4.1% lower (1.40 versus 1.46). This contrasted with the bootstrap standard error for the IPTW estimator which was closer to the adjusted regression standard error (both model based and bootstrap standard error), suggesting that the IPTW-W variance estimator may not be performing adequately in the small sample setting.

**Table 2:** Analysis of the ADAPT trial

Outcome	Analysis	TE	SE (bootstrap SE)	95% CI	*P*-value
Total SCORAD	Unadjusted	−7.86	3.75 (3.70)	−15.36 to − 0.36	0.040
	Adjusted - Regression	− 8.34	3.59 (3.62)	−15.53 to − 1.14	0.024
	Adjusted - IPTW	−8.27	3.43 (3.63)	−15.00 to − 1.54	0.016
EASI	Unadjusted	−5.62	3.36 (3.32)	−12.34 to 1.10	0.099
	Adjusted - Regression	−6.67	3.26 (3.29)	−13.21 to −0.13	0.046
	Adjusted - IPTW	−6.60	3.11 (3.26)	−12.69 to −0.50	0.034
(C)DLQI	Unadjusted	−3.30	1.48 (1.47)	−6.26 to −0.34	0.030
	Adjusted - Regression	−3.45	1.46 (1.47)	−6.38 to −0.53	0.022
	Adjusted - IPTW	−3.40	1.40 (1.49)	−6.15 to −0.65	0.015

Adjusted analysis includes adjustment for stratification factors Age (< 10 yrs., ≥10 yrs), IgE (≤1500, > 1500) and baseline value of associated outcome

Notably the degree of reduction in the estimated SE for IPTW versus regression adjustment was of a similar magnitude to the reduction obtained from adjusting for randomisation stratification factors and baseline outcome versus the unadjusted analysis. If we were to take a strict cut off as *p* < 0.05 to indicate significance, although we wouldn’t obtain any different conclusions from the two adjusted methods used here, in only slightly different cases the differences between the methods may result in different conclusions. For example, for EASI the *p*-value from the regression analysis was 0.046 versus 0.034 from IPTW; were the numbers marginally different alternative conclusions could have been reached. These results were consistent in sensitivity analysis where IgE was alternatively treated as a continuous variable (see Table C1 in Additional file 1).

## Discussion

We set out to explore the properties of IPTW using the estimated propensity score for baseline covariate adjustment in smaller population trial settings. With smaller sample sizes IPTW did not perform so well. The coverage of 95% CI’s was marginally below 95% for sample sizes of 100–150. For sample sizes < 100 the drop in coverage increased and was always significantly below 95%, indicating that the performance of IPTW is not optimal. The smallest sample size Williamson et al. explored the properties of IPTW for via simulation was *n* = 100 (50 per arm). Although with adjustment for 1 covariate they observed good performance of the IPTW-W variance estimate they too observed coverage significantly different to 95% for a continuous outcome when a larger number of 3 covariates were adjusted for, corresponding with our findings.

Subsequently we conducted adjusted analyses of three continuous outcomes from a paediatric eczema trial involving 60 participants using both IPTW and linear regression. The results confirmed that with smaller sample sizes there are differences between the linear regression variance estimator and the IPTW-W variance estimator. The IPTW-W variance estimate was lower than the estimated variance obtained for the treatment effect via linear regression for all three outcomes.

These results suggest in small trial settings with a continuous outcome there is a need for small-sample modifications for the IPTW estimator. Using the current large-sample version is likely to give over-precise results in very small samples. Fay and Graubard [28] showed that the sandwich variance estimator (which is used within IPTW) is biased downwards in small samples, which could also explain the reason of poor performance for IPTW. In larger samples IPTW using the propensity score method may however be a useful alternative. Williamson et al. demonstrated the large sample equivalence between the IPTW-W variance estimator and the analysis of covariance variance estimator theoretically and via simulation. Our simulation results using a larger sample of *n* = 150 and *n* = 200 reflect their findings. Thus we do not dispute that IPTW is a useful method for covariate adjustment in RCTs with large sample sizes. Moreover, when IPTW is used with large samples we have demonstrated how the bootstrap variance may be a simpler route to variance estimation, given this incorporates the estimation of the propensity score. When the bootstrap variance appropriately takes into account the estimation of the propensity score, it may be a more accessible way to compute the variance as the IPTW-W variance estimate involves more computational steps.

Examination of propensity score diagnostics confirmed excellent overlap across treatment arms on average in the simulation study, despite the small sample sizes, as expected due to randomisation. In the ADAPT case study (*n* = 62) the estimated propensity score distribution and overlap was also excellent. However, it cannot be ruled out that in future real life trial settings, despite randomisation, due to chance one may get extreme weights due to a lack of overlap in the estimated propensity score by treatment arm. This may result in an additional loss of precision in the small sample setting using such methods [29].

A strength of this study is the inclusion of a real life case study in addition to the simulations. The results from the eczema trial and the simulations correspond and lead us to our conclusions. We also carried out a variety of simulation scenarios with both continuous and binary covariates. All scenarios had at least 80% power reflecting typical RCT scenarios. Of course, as with any simulation study we were limited by the number of scenarios explored and our conclusions do not cover all settings and are based on an assumed correct normal outcome model. The EMA guidelines [6] recommend “no more than a few covariates should be included in the primary analysis” which was why we did not adjust for more than 6 covariates. Six was quite a large number anyway, particularly with sample sizes down to 40 corresponding to a low 6.7 SPV. With 80% power and two-sided 5% significance a sample size of 42 enables one to detect only a large standardised effect of 0.9SD. In smaller settings only very large effects could be detected. The results in Figs. 2 and 3 clearly show how the large sample equivalence of the variance estimator breaks down with smaller sample sizes.

Within our evaluations we concentrated on the analysis of a continuous outcome. We did not look at a binary or survival outcome since the statistical properties of covariate adjustment are different within these settings. The non-collapsibility of odds ratios and hazard ratios means that the estimated treatment effect will change in addition to the precision when baseline covariates are included within an adjusted logistic regression analysis. Whilst baseline adjustment also leads to increased power in logistic regression, this is not obtained via increasing the precision of the treatment effect [2]. Adjusted analysis via IPTW will preserve the marginal estimand and it has been shown to increase precision over an unadjusted analysis with large samples [14]. But based on our results for a continuous outcome we expect to observe similarly that IPTW does not perform so well with smaller samples with a binary or survival outcome. Large sample theory was used to derive the variance estimator for the IPTW treatment estimator in the binary outcome setting and previous simulations with a sample size of 100 (50 per arm) under-estimated the variance for a risk difference [14]. Further work is required to confirm the properties of IPTW estimators for a binary and survival outcome in small RCT settings. Valuable future work will also explore the use of small sample modifications to the IPTW estimator [28].

Throughout we compared the performance of IPTW using the propensity score against regression modelling for covariate adjustment. We chose to focus comparisons on regression analysis since this is the most commonly used method of adjustment and easily accessible. However alternative methods of adjustment exist, including performing a stratified analysis or using a semi-parametric estimator [4]. Other estimators are discussed in [30]. Further research is required to evaluate the performance of other methods of adjustment in smaller population RCT settings against IPTW.

## Conclusions

In conclusion with large samples, as shown by Williamson et al., IPTW using the estimated propensity score is unequivocally a useful alternative method for conducting baseline adjustment in RCTs. In larger sample settings we have demonstrated that the bootstrap variance is an alternative more accessible variance estimate to use within IPTW analysis. However we caution against the use of IPTW using the estimated propensity score, without small-sample modifications to the standard error, confidence interval and *p*-value calculation, in small sample settings when the sample size is less than 150 and particularly recommend against the use of IPTW without small-sample modifications when sample size is less than 100. A regression approach is preferable in such small sample settings.

## Supplementary information

**Additional file 1: Appendix A.** The IPTW treatment and variance estimator. **Appendix B.** Additional simulation methods and results.

**Additional file 2.** Stata code for case study: the ADAPT trial.

## Data Availability

The code used to analyse the case study is available in Additional file 2. The data from the case study comes from the ADAPT trial which is available from the corresponding author on reasonable request. The code used to generate and analyse datasets in the simulation study and for the case study are available from the corresponding author on reasonable request.

## Abbreviations

ADAPT: Atopic Dermatitis Anti-IgE Paediatric Trial
ANCOVA: Analysis of Covariance
(C) DLQI: The (Childrens) Dermatological Life Quality Index
CI: Confidence Interval
EASI: Eczema Area and Severity Index
EMA: European Medicines Agency
IPTW: Inverse Probability of Treatment Weighting
IPTW-W: Inverse Probability of Treatment Weighting variance estimator calculated using eq. 1 in Appendix A, Additional File 1
RCT: Randomised Controlled Trial
SCORAD: The total SCORing Atopic Dermatitis score
SE: Standard error
SPV: Subjects per variable
