# Supplementary material for: An evaluation of inverse probability weighting using the propensity score for baseline covariate adjustment in smaller population randomised controlled trials with a continuous outcome
Source: BMC Med Res Methodol. 2020 Mar 23;20:70. doi: 10.1186/s12874-020-00947-7 (PMC7092449; doi:10.1186/s12874-020-00947-7)
Supplement: Supplementary file 1 — Additional file 1: Appendix A. The IPTW treatment and variance estimator. Appendix B. Additional simulation methods and results. [file 12874_2020_947_MOESM1_ESM.docx]

# Additional File 1: An evaluation of inverse probability weighting using the propensity score for baseline covariate adjustment in smaller population randomised controlled trials

## Appendix A. The IPTW treatment and variance estimator

The IPTW treatment estimator for a continuous outcome $\hat{\mu}_{1}-\hat{\mu}_{0}$ is,

$$\hat{\mu}_{1}=\left( \sum_{i=1}^{n} \frac{Y_{i}Z_{i}}{\hat{e}_{i}} \right)\left( \sum_{i=n}^{n} \frac{Z_{i}}{\hat{e}_{i}} \right)^{-1} \hat{\mu}_{0}=\left( \sum_{i=1}^{n} \frac{Y_{i}\left( {1-Z}_{i} \right)}{\left( 1-\hat{e}_{i} \right)} \right)\left( \sum_{i=n}^{n} \frac{\left( 1-Z_{i} \right)}{\left( 1-\hat{e}_{i} \right)} \right)^{-1}$$

And the appropriate variance estimator for the IPTW treatment estimate ($\hat{Var}\left( \hat{\mu}_{1}-\hat{\mu}_{0} \right))$, as (equation 4 in [15]) , IPTW-W =

$n\hat{Var}\left( \hat{\mu}_{1}-\hat{\mu}_{0} \right)=\hat{V}_{un}-{\hat{\boldsymbol{v}}}^{T}\left( 2{\hat{\boldsymbol{M}}}_{1}-{\hat{\boldsymbol{M}}}_{2} \right)\hat{\boldsymbol{v}}$ (1)

where for a continuous outcome,

$$\hat{w}_{1}=\frac{1}{n}\sum_{i=1}^{n} \frac{Z_{i}}{\hat{e}_{i}}, \hat{w}_{0}=\frac{1}{n}\sum_{i=1}^{n} \frac{1-Z_{i}}{1-\hat{e}_{i}},$$

$$\hat{V}_{un}=\frac{1}{\hat{w}_{1}^{2}}\frac{1}{n}\sum_{i=1}^{n} \frac{\left( Y_{i}-\hat{\mu}_{1} \right)^{2}Z_{i}}{\hat{e}_{i}^{2}}+\frac{1}{\hat{w}_{0}^{2}}\frac{1}{n}\sum_{i=1}^{n} \frac{\left( Y_{i}-\hat{\mu}_{0} \right)^{2}\left( {1-Z}_{i} \right)}{\left( 1-\hat{e}_{i}^{2} \right)^{2}}$$

$$\hat{\boldsymbol{v}}=\frac{1}{\hat{w}_{1}}\frac{1}{n}\sum_{i=1}^{n} \frac{\boldsymbol{X}_{i}\left( Y_{i}-\hat{\mu}_{1} \right)Z_{i}\left( 1-\hat{e}_{i} \right)}{\hat{e}_{i}}+\frac{1}{\hat{w}_{0}}\frac{1}{n}\sum_{i=1}^{n} \frac{\boldsymbol{X}_{i}\left( Y_{i}-\hat{\mu}_{0} \right)\left( {1-Z}_{i} \right)\hat{e}_{i}}{\left( 1-\hat{e}_{i} \right)},$$

${\hat{\boldsymbol{M}}}_{1}=\left( \frac{1}{n}\sum_{i=1}^{n} \boldsymbol{X}_{i}\boldsymbol{X}_{i}^{T}\hat{e}_{i}\left( 1-\hat{e}_{i} \right) \right)^{-1}$and ${\hat{\boldsymbol{M}}}_{2}={\hat{\boldsymbol{M}}}_{1}\left( \frac{1}{n}\sum_{i=1}^{n} \boldsymbol{X}_{i}\boldsymbol{X}_{i}^{T}\left( Z_{i}-\hat{e}_{i} \right)^{2} \right){\hat{\boldsymbol{M}}}_{1}$

## Appendix B. Additional simulation methods and results

Additional methods

We also considered RCT scenarios with a mix of binary and continuous covariates. We generated three covariates from independent Bernoulli distribution (C1,C3, C5) each with parameter of 0.5 and three as independent standard normal variables with mean 0 and a variance of 1 (C2, C4, C6). A treatment arm indicator (Z) was again generated from a Bernouilli distribution with a probability of 0.5 and a normally distributed outcome Y, with mean E[Y]= 2*C1 + 2*C2 + 2*C3 + 2*C4 +2*C5 + 2*C6 + 5*Z and variance 5^2^ was then simulated. Therefore, for binary covariates a change in covariate value (from 0 to 1 for C1, C3, and C5) was associated with a 2 unit increase in outcome and for continuous covariates a one standard deviation increase in the variable was associated with a 2 unit increase in outcome. As in the continuous covariate case, sample sizes of 40-150 (in multiples of 10) and 200 were drawn, all trial scenarios had at least 80% power and a total of 2000 datasets were drawn for each scenario. Any model convergence issues or cases where the desired model could not be fit (e.g. with adjustment for all specified covariates due to a sparse data structure with binary covariates) were are also recorded.

In the smallest sample size setting (n=40) with a larger number of covariates (2, 4 and 6) there was one case where the analysis model of interest, including adjustment for all covariates could not be fitted. This was a result of a sparse data problem, indicating it would not be sensible to fit such a large model in such settings with either analysis method.

Additional results

Table B1: Simulation results – with adjustment for 1 to 6 continuous covariates

| N | Adjustment using multiple linear regression | | | | | | Adjustment using IPTW | | | | | |
| --- | --- | --- | --- | --- | --- | --- | --- | --- | --- | --- | --- | --- |
|  | Ave $\hat{\theta}$ | Mean %bias $\hat{\theta}$ | Mean $\hat{SE}$ | Empirical $SE\left( \hat{\theta} \right)$ | Mean $\hat{SE}$/$SE\left( \hat{\theta} \right)$ | Cov 95% CI | Ave $\hat{\theta}$ | Mean %bias $\hat{\theta}$ | Mean $\hat{SE}$ | Empirical $SE\left( \hat{\theta} \right)$ | Mean  $\hat{SE}$/ $SE\left( \hat{\theta} \right)$ | Cov 95% CI |
| Adjusted for C1 | | |  |  |  |  |  |  |  |  |  |  |
| 40 | 5.00 | 0.00 | 2.16 | 2.25 | 0.96 | 94.1 | 5.00 | -0.05 | 2.06 | 2.26 | 0.91 | 91.0 |
| 50 | 5.02 | 0.36 | 1.93 | 1.95 | 0.99 | 95.2 | 5.02 | 0.34 | 1.87 | 1.96 | 0.95 | 93.0 |
| 60 | 4.98 | -0.38 | 1.75 | 1.69 | 1.03 | 95.7 | 4.98 | -0.37 | 1.70 | 1.70 | 1.00 | 94.8 |
| 70 | 4.93 | -1.45 | 1.62 | 1.66 | 0.97 | 94.7 | 4.93 | -1.47 | 1.58 | 1.66 | 0.95 | 93.3 |
| 80 | 5.07 | 1.38 | 1.52 | 1.53 | 0.99 | 94.7 | 5.07 | 1.38 | 1.49 | 1.53 | 0.97 | 93.7 |
| 90 | 5.00 | -0.02 | 1.43 | 1.43 | 1.00 | 95.0 | 5.00 | 0.01 | 1.40 | 1.44 | 0.98 | 94.2 |
| 100 | 4.99 | -0.28 | 1.35 | 1.35 | 1.00 | 94.5 | 4.99 | -0.27 | 1.33 | 1.35 | 0.99 | 93.9 |
| 110 | 5.03 | 0.59 | 1.29 | 1.28 | 1.01 | 95.0 | 5.03 | 0.59 | 1.27 | 1.29 | 0.99 | 94.3 |
| 120 | 4.94 | -1.23 | 1.23 | 1.21 | 1.02 | 95.5 | 4.94 | -1.22 | 1.22 | 1.22 | 1.00 | 94.9 |
| 130 | 4.98 | -0.38 | 1.18 | 1.19 | 0.99 | 94.5 | 4.98 | -0.38 | 1.17 | 1.19 | 0.98 | 93.9 |
| 140 | 4.97 | -0.60 | 1.14 | 1.14 | 1.00 | 95.2 | 4.97 | -0.60 | 1.13 | 1.14 | 0.99 | 94.8 |
| 150 | 4.95 | -1.05 | 1.10 | 1.11 | 0.99 | 94.8 | 4.95 | -1.05 | 1.09 | 1.11 | 0.98 | 94.3 |
| 200 | 4.95 | -0.94 | 0.95 | 0.94 | 1.01 | 95.3 | 4.95 | -0.95 | 0.94 | 0.94 | 1.00 | 94.7 |
| Adjusted for C1 and C2 | | | |  |  |  |  |  |  |  |  |  |
| 40 | 4.88 | -2.33 | 2.09 | 2.13 | 0.98 | 93.9 | 4.88 | -2.40 | 1.96 | 2.16 | 0.90 | 90.8 |
| 50 | 5.02 | 0.50 | 1.87 | 1.88 | 0.99 | 95.2 | 5.02 | 0.46 | 1.78 | 1.91 | 0.93 | 92.2 |
| 60 | 5.04 | 0.74 | 1.70 | 1.74 | 0.98 | 94.5 | 5.03 | 0.63 | 1.63 | 1.75 | 0.93 | 93.2 |
| 70 | 5.00 | -0.03 | 1.56 | 1.58 | 0.99 | 95.3 | 4.99 | -0.12 | 1.51 | 1.58 | 0.96 | 94.0 |
| 80 | 4.98 | -0.31 | 1.45 | 1.47 | 0.99 | 94.5 | 4.98 | -0.36 | 1.41 | 1.48 | 0.95 | 93.5 |
| 90 | 4.99 | -0.20 | 1.37 | 1.41 | 0.97 | 94.3 | 4.99 | -0.25 | 1.33 | 1.41 | 0.95 | 93.2 |
| 100 | 4.99 | -0.13 | 1.30 | 1.30 | 0.99 | 94.8 | 4.99 | -0.14 | 1.27 | 1.31 | 0.97 | 94.1 |
| 110 | 5.03 | 0.66 | 1.24 | 1.24 | 1.00 | 94.3 | 5.03 | 0.65 | 1.21 | 1.24 | 0.98 | 93.6 |
| 120 | 4.93 | -1.38 | 1.18 | 1.16 | 1.02 | 94.7 | 4.93 | -1.40 | 1.16 | 1.17 | 1.00 | 94.1 |
| 130 | 4.97 | -0.54 | 1.13 | 1.15 | 0.99 | 94.0 | 4.97 | -0.51 | 1.11 | 1.15 | 0.97 | 93.4 |
| 140 | 4.97 | -0.58 | 1.09 | 1.10 | 0.99 | 94.6 | 4.97 | -0.59 | 1.08 | 1.10 | 0.98 | 94.4 |
| 150 | 4.95 | -0.93 | 1.06 | 1.06 | 0.99 | 94.6 | 4.95 | -0.94 | 1.04 | 1.06 | 0.98 | 94.3 |
| 200 | 5.00 | -0.07 | 0.91 | 0.91 | 1.00 | 95.0 | 5.00 | -0.06 | 0.90 | 0.91 | 0.99 | 94.2 |
| Adjusted for C1, C2, C3 and C4 | | | | |  |  |  |  |  |  |  |  |
| 40 | 4.92 | -1.67 | 1.93 | 1.96 | 0.98 | 94.2 | 4.92 | -1.63 | 1.78 | 2.03 | 0.88 | 89.1 |
| 50 | 4.99 | -0.25 | 1.71 | 1.73 | 0.99 | 95.9 | 4.99 | -0.26 | 1.61 | 1.78 | 0.91 | 92.3 |
| 60 | 5.04 | 0.85 | 1.55 | 1.59 | 0.98 | 94.9 | 5.04 | 0.75 | 1.47 | 1.62 | 0.91 | 92.3 |
| 70 | 5.00 | -0.01 | 1.42 | 1.42 | 1.00 | 95.0 | 4.99 | -0.18 | 1.36 | 1.43 | 0.96 | 93.8 |
| 80 | 5.00 | -0.02 | 1.32 | 1.32 | 1.01 | 95.2 | 4.99 | -0.11 | 1.27 | 1.33 | 0.95 | 93.8 |
| 90 | 4.99 | -0.29 | 1.24 | 1.28 | 0.97 | 95.1 | 4.98 | -0.32 | 1.20 | 1.28 | 0.93 | 93.4 |
| 100 | 4.99 | -0.26 | 1.17 | 1.17 | 1.00 | 95.3 | 4.98 | -0.31 | 1.14 | 1.19 | 0.96 | 94.0 |
| 110 | 5.03 | 0.67 | 1.12 | 1.11 | 1.01 | 95.0 | 5.03 | 0.63 | 1.09 | 1.11 | 0.98 | 93.8 |
| 120 | 4.94 | -1.13 | 1.07 | 1.07 | 1.00 | 95.2 | 4.94 | -1.14 | 1.04 | 1.08 | 0.97 | 94.5 |
| 130 | 5.00 | 0.00 | 1.02 | 1.05 | 0.98 | 94.5 | 5.00 | 0.02 | 1.00 | 1.05 | 0.95 | 93.4 |
| 140 | 4.98 | -0.32 | 0.99 | 0.99 | 1.00 | 94.1 | 4.98 | -0.37 | 0.97 | 0.99 | 0.98 | 93.6 |
| 150 | 4.97 | -0.66 | 0.95 | 0.96 | 0.99 | 95.0 | 4.97 | -0.65 | 0.93 | 0.96 | 0.97 | 94.0 |
| 200 | 5.01 | 0.26 | 0.82 | 0.82 | 1.00 | 95.4 | 5.01 | 0.26 | 0.81 | 0.82 | 0.98 | 94.6 |
| Adjusted for C1, C2, C3, C4, C5 and C6 | | | | | |  |  |  |  |  |  |  |
| 40 | 4.91 | -1.78 | 1.73 | 1.76 | 0.98 | 94.4 | 4.91 | -1.82 | 1.70 | 1.90 | 0.90 | 89.6 |
| 50 | 5.01 | 0.25 | 1.52 | 1.57 | 0.97 | 94.2 | 5.00 | 0.03 | 1.47 | 1.68 | 0.87 | 91.2 |
| 60 | 5.02 | 0.42 | 1.37 | 1.39 | 0.99 | 94.8 | 5.02 | 0.37 | 1.33 | 1.46 | 0.91 | 92.3 |
| 70 | 4.99 | -0.10 | 1.26 | 1.22 | 1.03 | 95.7 | 4.99 | -0.26 | 1.22 | 1.25 | 0.98 | 94.4 |
| 80 | 4.99 | -0.11 | 1.17 | 1.16 | 1.00 | 95.3 | 4.99 | -0.23 | 1.12 | 1.19 | 0.95 | 92.9 |
| 90 | 4.98 | -0.32 | 1.09 | 1.10 | 1.00 | 94.6 | 4.99 | -0.28 | 1.06 | 1.12 | 0.95 | 92.9 |
| 100 | 5.00 | -0.07 | 1.03 | 1.03 | 1.00 | 95.2 | 4.99 | -0.25 | 1.00 | 1.05 | 0.96 | 93.9 |
| 110 | 5.02 | 0.42 | 0.98 | 0.97 | 1.02 | 95.1 | 5.02 | 0.41 | 0.96 | 0.98 | 0.97 | 93.6 |
| 120 | 4.96 | -0.77 | 0.94 | 0.94 | 1.00 | 95.7 | 4.96 | -0.77 | 0.91 | 0.95 | 0.96 | 95.0 |
| 130 | 4.99 | -0.15 | 0.90 | 0.91 | 0.98 | 94.4 | 5.00 | -0.04 | 0.88 | 0.92 | 0.95 | 93.1 |
| 140 | 5.00 | -0.07 | 0.87 | 0.87 | 1.00 | 94.8 | 4.99 | -0.21 | 0.85 | 0.88 | 0.97 | 93.9 |
| 150 | 4.97 | -0.51 | 0.83 | 0.82 | 1.01 | 95.1 | 4.97 | -0.51 | 0.81 | 0.83 | 0.98 | 94.3 |
| 200 | 5.00 | 0.03 | 0.72 | 0.72 | 1.00 | 95.4 | 5.00 | 0.03 | 0.71 | 0.72 | 0.98 | 95.2 |

Table B2: Bootstrap standard error simulation results

| N | Adjustment using multiple linear regression | | | Adjustment using IPTW | | |
| --- | --- | --- | --- | --- | --- | --- |
|  | Empirical $SE\left( \hat{\theta} \right)$ | Mean$\hat{SE}_{boot}$ | Mean $\hat{SE}_{boot}/S\left( \hat{\theta} \right)$ | Empirical $SE\left( \hat{\theta} \right)$ | Mean$\hat{SE}_{boot}$ | Mean $\hat{SE}$/ $SE\left( \hat{\theta} \right)$ |
| Adjusted for C1 | | |  |  |  |  |
| 40 | 2.25 | 2.15 | 0.95 | 2.26 | 2.16 | 0.96 |
| 50 | 1.95 | 1.92 | 0.98 | 1.96 | 1.93 | 0.98 |
| 60 | 1.69 | 1.74 | 1.03 | 1.70 | 1.74 | 1.03 |
| 70 | 1.66 | 1.61 | 0.97 | 1.66 | 1.61 | 0.97 |
| 80 | 1.53 | 1.51 | 0.99 | 1.53 | 1.51 | 0.99 |
| 90 | 1.43 | 1.42 | 0.99 | 1.44 | 1.42 | 0.99 |
| 100 | 1.35 | 1.35 | 1.00 | 1.35 | 1.35 | 1.00 |
| 110 | 1.28 | 1.28 | 1.00 | 1.29 | 1.28 | 1.00 |
| 120 | 1.21 | 1.23 | 1.01 | 1.22 | 1.23 | 1.01 |
| 130 | 1.19 | 1.18 | 0.99 | 1.19 | 1.18 | 0.99 |
| 140 | 1.14 | 1.14 | 1.00 | 1.14 | 1.14 | 1.00 |
| 150 | 1.11 | 1.10 | 0.99 | 1.11 | 1.10 | 0.99 |
| 200 | 0.94 | 0.95 | 1.00 | 0.94 | 0.95 | 1.00 |
| Adjusted for C1 and C2 | | |  |  |  |  |
| 40 | 2.13 | 2.09 | 0.98 | 2.16 | 2.14 | 0.99 |
| 50 | 1.88 | 1.86 | 0.99 | 1.91 | 1.89 | 0.99 |
| 60 | 1.74 | 1.69 | 0.97 | 1.75 | 1.72 | 0.98 |
| 70 | 1.58 | 1.56 | 0.99 | 1.58 | 1.57 | 1.00 |
| 80 | 1.47 | 1.45 | 0.98 | 1.48 | 1.46 | 0.98 |
| 90 | 1.41 | 1.36 | 0.97 | 1.41 | 1.37 | 0.97 |
| 100 | 1.30 | 1.29 | 0.99 | 1.31 | 1.30 | 0.99 |
| 110 | 1.24 | 1.23 | 1.00 | 1.24 | 1.24 | 1.00 |
| 120 | 1.16 | 1.18 | 1.01 | 1.17 | 1.18 | 1.01 |
| 130 | 1.15 | 1.13 | 0.98 | 1.15 | 1.13 | 0.98 |
| 140 | 1.10 | 1.09 | 0.99 | 1.10 | 1.09 | 0.99 |
| 150 | 1.06 | 1.05 | 0.99 | 1.06 | 1.05 | 0.99 |
| 200 | 0.91 | 0.91 | 1.00 | 0.91 | 0.91 | 1.00 |
| Adjusted for C1, C2, C3 and C4 | | | |  |  |  |
| 40 | 1.96 | 1.97 | 1.00 | 2.03 | 2.19 | 1.08 |
| 50 | 1.73 | 1.73 | 1.00 | 1.78 | 1.89 | 1.06 |
| 60 | 1.59 | 1.56 | 0.98 | 1.62 | 1.67 | 1.03 |
| 70 | 1.42 | 1.43 | 1.01 | 1.43 | 1.51 | 1.06 |
| 80 | 1.32 | 1.33 | 1.01 | 1.33 | 1.37 | 1.03 |
| 90 | 1.28 | 1.24 | 0.97 | 1.28 | 1.28 | 0.99 |
| 100 | 1.17 | 1.17 | 1.00 | 1.19 | 1.20 | 1.01 |
| 110 | 1.11 | 1.12 | 1.00 | 1.11 | 1.14 | 1.02 |
| 120 | 1.07 | 1.07 | 1.00 | 1.08 | 1.08 | 1.01 |
| 130 | 1.05 | 1.02 | 0.97 | 1.05 | 1.03 | 0.98 |
| 140 | 0.99 | 0.98 | 1.00 | 0.99 | 1.00 | 1.01 |
| 150 | 0.96 | 0.95 | 0.99 | 0.96 | 0.96 | 0.99 |
| 200 | 0.82 | 0.82 | 1.00 | 0.82 | 0.82 | 1.00 |
| Adjusted for C1, C2, C3, C4, C5 and C6 | | | |  |  |  |
| 40 | 1.76 | 1.83 | 1.04 | 1.90 | 2.37 | 1.25 |
| 50 | 1.57 | 1.57 | 1.00 | 1.68 | 1.99 | 1.18 |
| 60 | 1.39 | 1.40 | 1.01 | 1.46 | 1.71 | 1.17 |
| 70 | 1.22 | 1.27 | 1.04 | 1.25 | 1.51 | 1.21 |
| 80 | 1.16 | 1.18 | 1.01 | 1.19 | 1.33 | 1.12 |
| 90 | 1.10 | 1.10 | 1.00 | 1.12 | 1.21 | 1.08 |
| 100 | 1.03 | 1.04 | 1.00 | 1.05 | 1.12 | 1.07 |
| 110 | 0.97 | 0.99 | 1.02 | 0.98 | 1.05 | 1.07 |
| 120 | 0.94 | 0.94 | 1.00 | 0.95 | 0.99 | 1.04 |
| 130 | 0.91 | 0.90 | 0.98 | 0.92 | 0.94 | 1.02 |
| 140 | 0.87 | 0.87 | 0.99 | 0.88 | 0.90 | 1.03 |
| 150 | 0.82 | 0.83 | 1.01 | 0.83 | 0.86 | 1.03 |
| 200 | 0.72 | 0.72 | 1.00 | 0.72 | 0.73 | 1.02 |

Table B3: Simulation results with binary (C1, C3 and C5) and continuous (C2, C4, and C6) covariates – with adjustment for 1 to 6 continuous covariates

| N | Adjustment using multiple linear regression | | | | | | Adjustment using IPTW | | | | | |
| --- | --- | --- | --- | --- | --- | --- | --- | --- | --- | --- | --- | --- |
|  | Ave $\hat{\theta}$ | Mean %bias $\hat{\theta}$ | Mean $\hat{SE}$ | Empirical $SE\left( \hat{\theta} \right)$ | Mean $\hat{SE}$/$SE\left( \hat{\theta} \right)$ | Cov 95% CI | Ave $\hat{\theta}$ | Mean %bias $\hat{\theta}$ | Mean $\hat{SE}$ | Empirical $SE\left( \hat{\theta} \right)$ | Mean  $\hat{SE}$/ $SE\left( \hat{\theta} \right)$ | Cov 95% CI |
| Adjusted for C1 | | |  |  |  |  |  |  |  |  |  |  |
| 40 | 5.00 | 0.05 | 2.02 | 2.00 | 1.01 | 95.6 | 5.00 | 0.06 | 1.93 | 2.00 | 0.96 | 93.6 |
| 50 | 4.97 | -0.54 | 1.79 | 1.80 | 0.99 | 95.2 | 4.97 | -0.59 | 1.73 | 1.80 | 0.96 | 93.5 |
| 60 | 4.99 | -0.30 | 1.64 | 1.67 | 0.99 | 95.2 | 4.99 | -0.24 | 1.60 | 1.67 | 0.96 | 93.5 |
| 70 | 5.01 | 0.22 | 1.51 | 1.53 | 0.98 | 95.0 | 5.01 | 0.21 | 1.47 | 1.54 | 0.96 | 93.6 |
| 80 | 4.98 | -0.34 | 1.41 | 1.43 | 0.99 | 94.7 | 4.98 | -0.32 | 1.38 | 1.43 | 0.97 | 93.8 |
| 90 | 4.98 | -0.34 | 1.33 | 1.35 | 0.98 | 94.6 | 4.98 | -0.34 | 1.30 | 1.35 | 0.96 | 94.0 |
| 100 | 5.00 | 0.01 | 1.26 | 1.25 | 1.00 | 94.9 | 5.00 | 0.00 | 1.24 | 1.25 | 0.99 | 94.4 |
| 110 | 4.99 | -0.10 | 1.20 | 1.22 | 0.98 | 95.0 | 4.99 | -0.11 | 1.18 | 1.22 | 0.97 | 94.3 |
| 120 | 5.01 | 0.15 | 1.15 | 1.14 | 1.01 | 94.7 | 5.01 | 0.15 | 1.14 | 1.14 | 1.00 | 94.2 |
| 130 | 4.96 | -0.80 | 1.10 | 1.09 | 1.01 | 94.5 | 4.96 | -0.81 | 1.09 | 1.09 | 1.00 | 93.7 |
| 140 | 4.98 | -0.33 | 1.06 | 1.07 | 0.99 | 94.8 | 4.98 | -0.33 | 1.05 | 1.07 | 0.98 | 94.4 |
| 150 | 4.97 | -0.64 | 1.02 | 1.03 | 1.00 | 94.9 | 4.97 | -0.63 | 1.01 | 1.03 | 0.99 | 94.4 |
| 200 | 4.97 | -0.60 | 0.89 | 0.91 | 0.98 | 94.5 | 4.97 | -0.59 | 0.88 | 0.91 | 0.97 | 94.2 |
| Adjusted for C1 and C2 | | | |  |  |  |  |  |  |  |  |  |
| 40* | 5.09 | 1.74 | 1.93 | 1.96 | 0.99 | 95.4 | 5.09 | 1.85 | 1.81 | 1.97 | 0.91 | 92.2 |
| 50 | 4.99 | -0.26 | 1.71 | 1.67 | 1.03 | 95.6 | 4.98 | -0.32 | 1.63 | 1.68 | 0.97 | 93.9 |
| 60 | 5.01 | 0.19 | 1.57 | 1.58 | 0.99 | 95.0 | 5.01 | 0.16 | 1.51 | 1.58 | 0.95 | 93.3 |
| 70 | 4.99 | -0.26 | 1.45 | 1.43 | 1.01 | 94.7 | 4.98 | -0.30 | 1.40 | 1.43 | 0.98 | 93.3 |
| 80 | 5.03 | 0.68 | 1.34 | 1.37 | 0.98 | 94.4 | 5.03 | 0.63 | 1.31 | 1.38 | 0.95 | 93.1 |
| 90 | 5.03 | 0.62 | 1.26 | 1.24 | 1.02 | 95.7 | 5.03 | 0.65 | 1.23 | 1.24 | 0.99 | 94.7 |
| 100 | 5.02 | 0.38 | 1.20 | 1.18 | 1.02 | 95.8 | 5.02 | 0.38 | 1.17 | 1.18 | 1.00 | 95.1 |
| 110 | 5.02 | 0.33 | 1.14 | 1.14 | 1.01 | 95.1 | 5.02 | 0.32 | 1.12 | 1.13 | 0.99 | 94.4 |
| 120 | 4.97 | -0.55 | 1.09 | 1.12 | 0.98 | 94.4 | 4.97 | -0.54 | 1.07 | 1.12 | 0.96 | 93.9 |
| 130 | 4.95 | -1.08 | 1.05 | 1.03 | 1.02 | 94.9 | 4.95 | -1.08 | 1.03 | 1.03 | 1.00 | 94.5 |
| 140 | 4.97 | -0.60 | 1.01 | 1.00 | 1.01 | 95.5 | 4.97 | -0.60 | 0.99 | 1.00 | 0.99 | 95.1 |
| 150 | 5.02 | 0.45 | 0.97 | 0.98 | 0.99 | 94.2 | 5.02 | 0.46 | 0.96 | 0.98 | 0.98 | 93.8 |
| 200 | 4.98 | -0.31 | 0.84 | 0.84 | 1.01 | 95.2 | 4.98 | -0.31 | 0.83 | 0.84 | 0.99 | 95.0 |
| Adjusted for C1, C2, C3 and C4 | | | | |  |  |  |  |  |  |  |  |
| 40* | 4.98 | -0.41 | 1.83 | 1.84 | 1.00 | 95.8 | 4.99 | -0.21 | 1.66 | 1.91 | 0.87 | 90.9 |
| 50 | 4.99 | -0.16 | 1.62 | 1.60 | 1.01 | 94.7 | 4.98 | -0.37 | 1.50 | 1.62 | 0.93 | 92.0 |
| 60 | 4.99 | -0.16 | 1.48 | 1.49 | 1.00 | 95.1 | 4.99 | -0.13 | 1.39 | 1.50 | 0.93 | 92.4 |
| 70 | 5.00 | 0.01 | 1.36 | 1.36 | 1.00 | 95.2 | 5.01 | 0.11 | 1.29 | 1.37 | 0.94 | 92.9 |
| 80 | 5.02 | 0.40 | 1.26 | 1.28 | 0.99 | 94.1 | 5.02 | 0.32 | 1.21 | 1.29 | 0.94 | 92.7 |
| 90 | 4.99 | -0.16 | 1.18 | 1.23 | 0.96 | 94.5 | 5.00 | -0.09 | 1.14 | 1.23 | 0.92 | 92.9 |
| 100 | 5.02 | 0.38 | 1.12 | 1.13 | 0.99 | 95.1 | 5.02 | 0.37 | 1.08 | 1.13 | 0.96 | 94.1 |
| 110 | 5.01 | 0.14 | 1.07 | 1.07 | 1.00 | 94.9 | 5.01 | 0.14 | 1.04 | 1.07 | 0.97 | 93.9 |
| 120 | 4.97 | -0.54 | 1.02 | 1.04 | 0.98 | 94.4 | 4.97 | -0.51 | 0.99 | 1.04 | 0.96 | 93.6 |
| 130 | 4.97 | -0.58 | 0.98 | 0.96 | 1.03 | 95.7 | 4.97 | -0.65 | 0.96 | 0.96 | 1.00 | 94.6 |
| 140 | 4.97 | -0.69 | 0.94 | 0.94 | 1.00 | 95.3 | 4.97 | -0.65 | 0.92 | 0.94 | 0.98 | 94.2 |
| 150 | 5.03 | 0.56 | 0.91 | 0.92 | 0.99 | 94.5 | 5.03 | 0.59 | 0.89 | 0.92 | 0.97 | 94.1 |
| 200 | 4.98 | -0.34 | 0.78 | 0.77 | 1.01 | 95.6 | 4.98 | -0.33 | 0.77 | 0.77 | 1.00 | 94.9 |
| Adjusted for C1, C2, C3, C4, C5 and C6 | | | | | |  |  |  |  |  |  |  |
| 40* | 5.02 | 0.38 | 1.73 | 1.75 | 0.99 | 94.9 | 5.02 | 0.49 | 1.60 | 1.90 | 0.84 | 89.6 |
| 50 | 5.00 | -0.09 | 1.52 | 1.49 | 1.02 | 95.3 | 5.00 | -0.07 | 1.40 | 1.54 | 0.91 | 91.3 |
| 60 | 5.00 | -0.03 | 1.37 | 1.39 | 0.99 | 94.1 | 5.00 | 0.06 | 1.29 | 1.45 | 0.89 | 91.1 |
| 70 | 5.02 | 0.31 | 1.26 | 1.25 | 1.00 | 95.0 | 5.02 | 0.40 | 1.18 | 1.29 | 0.92 | 91.9 |
| 80 | 5.02 | 0.47 | 1.17 | 1.18 | 0.99 | 94.7 | 5.02 | 0.37 | 1.11 | 1.20 | 0.93 | 92.3 |
| 90 | 4.98 | -0.42 | 1.09 | 1.12 | 0.98 | 94.8 | 4.98 | -0.47 | 1.04 | 1.13 | 0.92 | 93.1 |
| 100 | 5.05 | 0.94 | 1.03 | 1.05 | 0.99 | 94.7 | 5.04 | 0.89 | 1.00 | 1.05 | 0.95 | 93.2 |
| 110 | 5.00 | 0.06 | 0.98 | 0.99 | 0.99 | 94.8 | 5.00 | 0.03 | 0.95 | 1.00 | 0.95 | 93.4 |
| 120 | 4.98 | -0.32 | 0.94 | 0.94 | 0.99 | 95.7 | 4.98 | -0.34 | 0.91 | 0.95 | 0.96 | 94.3 |
| 130 | 4.98 | -0.40 | 0.90 | 0.88 | 1.02 | 95.7 | 4.98 | -0.39 | 0.88 | 0.89 | 0.99 | 94.2 |
| 140 | 4.96 | -0.75 | 0.87 | 0.86 | 1.01 | 95.5 | 4.97 | -0.69 | 0.84 | 0.86 | 0.98 | 94.7 |
| 150 | 5.02 | 0.42 | 0.84 | 0.83 | 1.00 | 95.2 | 5.02 | 0.46 | 0.82 | 0.84 | 0.97 | 93.8 |
| 200 | 4.99 | -0.15 | 0.72 | 0.71 | 1.01 | 95.6 | 4.99 | -0.14 | 0.70 | 0.71 | 0.99 | 95.1 |

*For n=40 with 2, 4 and 6 covariates model did not converge for 1 simulated data set. Thus results presented for 1999 simulations.

Table B4: Bootstrap standard error simulation results with binary (C1, C3 and C5) and continuous (C2, C4, and C6) covariates

| N | Adjustment using multiple linear regression | | | Adjustment using IPTW | | |
| --- | --- | --- | --- | --- | --- | --- |
|  | Mean $\hat{SE}$ | Empirical $SE\left( \hat{\theta} \right)$ | Mean $\hat{SE}$/ $SE\left( \hat{\theta} \right)$ | Mean $\hat{SE}$ | Empirical $SE\left( \hat{\theta} \right)$ | Mean $\hat{SE}$/ $SE\left( \hat{\theta} \right)$ |
| Adjusted for C1 | | |  |  |  |  |
| 40 | 2.00 | 2.00 | 1.00 | 2.00 | 2.00 | 1.00 |
| 50 | 1.78 | 1.80 | 0.98 | 1.78 | 1.80 | 0.99 |
| 60 | 1.63 | 1.67 | 0.98 | 1.63 | 1.67 | 0.98 |
| 70 | 1.50 | 1.53 | 0.98 | 1.50 | 1.54 | 0.98 |
| 80 | 1.40 | 1.43 | 0.98 | 1.40 | 1.43 | 0.98 |
| 90 | 1.32 | 1.35 | 0.98 | 1.32 | 1.35 | 0.98 |
| 100 | 1.25 | 1.25 | 1.00 | 1.25 | 1.25 | 1.00 |
| 110 | 1.19 | 1.22 | 0.98 | 1.19 | 1.22 | 0.98 |
| 120 | 1.15 | 1.14 | 1.01 | 1.15 | 1.14 | 1.01 |
| 130 | 1.10 | 1.09 | 1.01 | 1.10 | 1.09 | 1.01 |
| 140 | 1.06 | 1.07 | 0.99 | 1.06 | 1.07 | 0.99 |
| 150 | 1.02 | 1.03 | 0.99 | 1.02 | 1.03 | 0.99 |
| 200 | 0.89 | 0.91 | 0.97 | 0.89 | 0.91 | 0.97 |
| Adjusted for C1 and C2 | | |  |  |  |  |
| 40 | 1.92 | 1.96 | 0.98 | 1.96 | 1.97 | 0.99 |
| 50 | 1.71 | 1.67 | 1.02 | 1.73 | 1.68 | 1.03 |
| 60 | 1.56 | 1.58 | 0.99 | 1.57 | 1.58 | 1.00 |
| 70 | 1.44 | 1.43 | 1.01 | 1.45 | 1.43 | 1.01 |
| 80 | 1.34 | 1.37 | 0.98 | 1.34 | 1.38 | 0.98 |
| 90 | 1.26 | 1.24 | 1.01 | 1.26 | 1.24 | 1.02 |
| 100 | 1.20 | 1.18 | 1.02 | 1.20 | 1.18 | 1.02 |
| 110 | 1.14 | 1.14 | 1.00 | 1.14 | 1.13 | 1.00 |
| 120 | 1.09 | 1.12 | 0.97 | 1.09 | 1.12 | 0.97 |
| 130 | 1.05 | 1.03 | 1.02 | 1.05 | 1.03 | 1.02 |
| 140 | 1.01 | 1.00 | 1.00 | 1.01 | 1.00 | 1.00 |
| 150 | 0.97 | 0.98 | 0.99 | 0.97 | 0.98 | 0.99 |
| 200 | 0.84 | 0.84 | 1.00 | 0.84 | 0.84 | 1.00 |
| Adjusted for C1, C2, C3 and C4 | | | |  |  |  |
| 40 | 1.87 | 1.84 | 1.01 | 2.03 | 1.91 | 1.07 |
| 50 | 1.63 | 1.60 | 1.02 | 1.74 | 1.62 | 1.08 |
| 60 | 1.49 | 1.49 | 1.00 | 1.55 | 1.50 | 1.04 |
| 70 | 1.36 | 1.36 | 1.00 | 1.40 | 1.37 | 1.03 |
| 80 | 1.26 | 1.28 | 0.99 | 1.30 | 1.29 | 1.01 |
| 90 | 1.18 | 1.23 | 0.96 | 1.20 | 1.23 | 0.98 |
| 100 | 1.12 | 1.13 | 0.99 | 1.14 | 1.13 | 1.00 |
| 110 | 1.07 | 1.07 | 1.00 | 1.08 | 1.07 | 1.01 |
| 120 | 1.02 | 1.04 | 0.98 | 1.03 | 1.04 | 0.99 |
| 130 | 0.98 | 0.96 | 1.02 | 0.99 | 0.96 | 1.03 |
| 140 | 0.94 | 0.94 | 1.00 | 0.95 | 0.94 | 1.00 |
| 150 | 0.91 | 0.92 | 0.99 | 0.91 | 0.92 | 0.99 |
| 200 | 0.78 | 0.77 | 1.01 | 0.78 | 0.77 | 1.01 |
| Adjusted for C1, C2, C3, C4, C5 and C6 | | | |  |  |  |
| 40 | 1.81 | 1.75 | 1.03 | 2.22 | 1.90 | 1.17 |
| 50 | 1.56 | 1.49 | 1.04 | 1.86 | 1.54 | 1.20 |
| 60 | 1.40 | 1.39 | 1.00 | 1.61 | 1.45 | 1.11 |
| 70 | 1.26 | 1.25 | 1.01 | 1.41 | 1.29 | 1.09 |
| 80 | 1.18 | 1.18 | 1.00 | 1.28 | 1.20 | 1.07 |
| 90 | 1.10 | 1.12 | 0.98 | 1.16 | 1.13 | 1.03 |
| 100 | 1.04 | 1.05 | 0.99 | 1.09 | 1.05 | 1.04 |
| 110 | 0.99 | 0.99 | 1.00 | 1.02 | 1.00 | 1.03 |
| 120 | 0.94 | 0.94 | 1.00 | 0.97 | 0.95 | 1.02 |
| 130 | 0.90 | 0.88 | 1.03 | 0.93 | 0.89 | 1.05 |
| 140 | 0.87 | 0.86 | 1.01 | 0.88 | 0.86 | 1.03 |
| 150 | 0.84 | 0.83 | 1.00 | 0.85 | 0.84 | 1.02 |
| 200 | 0.72 | 0.71 | 1.01 | 0.72 | 0.71 | 1.02 |

## Appendix C. Sensitivity analysis of the ADAPT trial

Table C1: Sensitivity analysis of the ADAPT trial with a) adjustment for IgE as a continuous variable and b) exclusion of IgE

| Outcome | Analysis | TE | SE (bootstrap SE) | 95% CI | P-value |
| --- | --- | --- | --- | --- | --- |
| 1. Adjusting for IgE as a continuous variable | | | | |  |
| Total SCORAD | Unadjusted | -7.86 | 3.75 (3.70) | -15.36 to -0.36 | 0.040 |
|  | Adjusted using Regression | -8.07 | 3.62 (3.70) | -15.33 to -0.81 | 0.030 |
|  | Adjusted using IPTW | -7.94 | 3.57 (3.94) | -14.93 to -0.95 | 0.026 |
| EASI | Unadjusted | -5.62 | 3.36 (3.32) | -12.34 to 1.10 | 0.099 |
|  | Adjusted using Regression | -6.63 | 3.28 (3.31) | -13.20 to -0.07 | 0.048 |
|  | Adjusted using IPTW | -6.58 | 3.19 (3.37) | -12.83 to -0.33 | 0.039 |
| CDLQI | Unadjusted | -3.30 | 1.48 (1.47) | -6.26 to -0.34 | 0.030 |
|  | Adjusted using Regression | -3.19 | 1.49 (1.47) | -6.18 to -0.20 | 0.037 |
|  | Adjusted using IPTW | -3.23 | 1.42 (1.48) | -6.01 to -0.46 | 0.023 |
| b) Excluding IgE | | |  |  |  |
| Total SCORAD | Unadjusted | -7.86 | 3.75 (3.70) | -15.36 to -0.36 | 0.040 |
|  | Adjusted using Regression | -8.09 | 3.57 (3.61) | -15.24 to -0.94 | 0.027 |
|  | Adjusted using IPTW | -8.09 | 3.46 (3.61) | -14.88 to -1.30 | 0.020 |
| EASI | Unadjusted | -5.62 | 3.36 (3.32) | -12.34 to 1.10 | 0.099 |
|  | Adjusted using Regression | -6.41 | 3.25 (3.27) | -12.93 to 0.11 | 0.054 |
|  | Adjusted using IPTW | -6.39 | 3.12 (3.24) | -12.57 to -0.22 | 0.042 |
| CDLQI | Unadjusted | -3.30 | 1.48 (1.47) | -6.26 to -0.34 | 0.030 |
|  | Adjusted using Regression | -3.26 | 1.48 (1.50) | -6.22 to -0.31 | 0.031 |
|  | Adjusted using IPTW | -3.26 | 1.44 (1.48) | -6.08 to -0.45 | 0.023 |
