# Supplementary material for: An evaluation of inverse probability weighting using the propensity score for baseline covariate adjustment in smaller population randomised controlled trials with a continuous outcome
Source: BMC Med Res Methodol. 2020 Mar 23;20:70. doi: 10.1186/s12874-020-00947-7 (PMC7092449; doi:10.1186/s12874-020-00947-7)
Supplement: Supplementary file 2 — Additional file 2. Stata code for case study: the ADAPT trial. [file 12874_2020_947_MOESM2_ESM.docx]

**/*Code for case study: the ADAPT trial**

Stata code is provided for the analyses of the EASI outcome in the ADAPT case study within “An evaluation of inverse probability weighting using the propensity score for baseline covariate adjustment in smaller population randomised controlled trials.” The data are assumed to be in a file case_study.dta with one record per randomised individual. EASI_w24 is the EASI outcome at week 24, EASI_1 in baseline EASI score, treat in the randomised assignment to 0=placebo or 1 =omalizumab, agestrat is the baseline age, IgEstrat is the baseline IgE and package and id is the unique participant identifier. The same code was used to analyse the total SCORAD and (C)DLQI – replacing EASI_w24 and EASI_1 with the alternative outcome measures.*/

set seed 333

use case_study, clear

regress EASI_w24 treat

local treat = _b[treat]

postfile un_1 Treat using un_1, replace

forvalues i=1(1)10000{

preserve

bsample

regress EASI_w24 treat

local treat_boot=_b[treat]

restore

post un_1 (`treat_boot')

}

postclose un_1

bstat using un_1 , stat(`treat')

mat list e(V)

/*Adjusted via regression for binary IgEstrat */

regress EASI_w24 treat agestrat IgEstrat EASI_1

local treat = _b[treat]

postfile ad_1 Treat using ad_1, replace

forvalues i=1(1)10000{

preserve

bsample

regress EASI_w24 treat agestrat IgEstrat EASI_1

local treat_boot=_b[treat]

restore

post ad_1 (`treat_boot')

}

postclose ad_1

bstat using ad_1 , stat(`treat')

mat list e(V)

/*Adjusted via regression for continuous IgEstrat */

regress EASI_w24 treat agestrat IgEbaseline1 EASI_1

local treat = _b[treat]

postfile ad_2 Treat using ad_2, replace

forvalues i=1(1)10000{

preserve

bsample

regress EASI_w24 treat agestrat IgEbaseline1 EASI_1

local treat_boot=_b[treat]

restore

post ad_2 (`treat_boot')

}

postclose ad_2

bstat using ad_2 , stat(`treat')

mat list e(V)

/*Adjusted via IPTW using the estimated propensity score for binary IgEstrat */

logit treat agestrat IgEstrat EASI_1 if EASI_w24!=.

predict ps

gen ipw=1/ps

replace ipw=1/(1-ps) if (treat==0 )

*Mu_1:

gen P1=(EASI_w24*treat)/ps

gen P2=treat/ps

summ P1

local C1 = r(sum)

summ P2

local C2 = r(sum)

local C2=`C2'^(-1)

scalar mu_1=`C1'*`C2'

di mu_1

*Mu_0:

gen P3=(EASI_w24*(1-treat))/(1-ps)

gen P4=(1-treat)/(1-ps)

summ P3

local C3 = r(sum)

summ P4

local C4 = r(sum)

local C4=`C4'^(-1)

scalar mu_0=`C3'*`C4'

di mu_0

di mu_0-mu_1

regress EASI_w24 treat [pweight=ipw]

/*Note: variance estimate is not correct from the above model*/

*Calculating IPTW-W variance estimator:

gen int_1=treat/ps

summ int_1

scalar w1 = (1/60)*r(sum)

gen int_2=(1-treat)/(1-ps)

summ int_2

scalar w0 = (1/60)*r(sum)

scalar K01 = 1

scalar K11 = 1

gen int_3=(((EASI_w24-mu_1)^2)*treat)/(ps*ps)

summ int_3

scalar int_3_sum=r(sum)

gen int_4=(((EASI_w24-mu_0)^2)*(1-treat))/((1-ps)*(1-ps))

summ int_4

scalar int_4_sum=r(sum)

scalar V_un = (((K11*K11)/(w1*w1))*(1/60)*int_3_sum)+((((K01*K01)/(w0*w0)))*(1/60)*int_4_sum)

di V_un

forvalues i=1/60{

local cov1 = EASI_1 in `i'

local cov2 = IgEstrat in `i'

local cov3 = agestrat in `i'

mat define x`i' = [1,`cov1' , `cov2', `cov3']'

local mult`i' = ((EASI_w24-mu_1)*treat*(1-ps))/ps in `i'

mat define x1_`i'=x`i'*`mult`i''

}

mat define x1_sum = x1_1

forvalues i=2/60{

mat define x1_sum= x1_sum+x1_`i'

}

forvalues i=1/60{

local cov1 = EASI_1 in `i'

local cov2 = IgEstrat in `i'

local cov3 = agestrat in `i'

mat define x`i' = [1,`cov1' , `cov2', `cov3']'

local Tmult`i' = ((EASI_w24-mu_0)*(1-treat)*(ps))/(1-ps) in `i'

mat define x2_`i'=x`i'*`Tmult`i''

}

mat define x2_sum = x2_1

forvalues i=2/60{

mat define x2_sum= x2_sum+x2_`i'

}

mat define v_hat = ((((K11/w1)*(1/60)))*x1_sum) + ((((K01)/w0*(1/60)))*x2_sum)

mat list v_hat

forvalues i=1/60{

local cov1 = EASI_1 in `i'

local cov2 = IgEstrat in `i'

local cov3 = agestrat in `i'

mat define x`i' = [1,`cov1' , `cov2', `cov3']'

local Mult_`i' = (ps)*(1-ps) in `i'

mat define x_x_`i'=x`i'*x`i''

mat define ext_`i' = x_x_`i'*`Mult_`i''

}

mat define M1_sum = ext_1

forvalues i=2/60{

mat define M1_sum= M1_sum+ext_`i'

}

mat define M1_int = ((1/60)*M1_sum)

mat define M1=inv(M1_int)

forvalues i=1/60{

local cov1 = EASI_1 in `i'

local cov2 = IgEstrat in `i'

local cov3 = agestrat in `i'

mat define x`i' = [1,`cov1' , `cov2', `cov3']'

local Mult_`i' = (treat -ps)^2 in `i'

mat define x_x_`i'=x`i'*x`i''

mat define ext_`i' = x_x_`i'*`Mult_`i''

}

mat define M2_sum = ext_1

forvalues i=2/60{

mat define M2_sum= M2_sum+ext_`i'

}

mat define M2 = M1*((1/60)*M2_sum)*M1

mat define nvar_delta = V_un - (v_hat'*((2*M1)-M2)*v_hat)

mat list nvar_delta

di nvar_delta[1,1]/60

di sqrt(nvar_delta[1,1]/60)

*CI using normal distribution:

di -6.599213+1.96* 3.1096958

di -6.599213-1.96* 3.1096958

*p-value:

local t= -6.599213/3.1096958

di 2*(1-normal(abs(`t')))

drop ps

drop ipw

local treat=mu_0-mu_1

postfile ps_1 Treat using ps_1, replace

forvalues i=1(1)10000{

preserve

bsample

logit treat agestrat IgEstrat EASI_1 if EASI_w24!=.

predict ps

gen ipw=1/ps

replace ipw=1/(1-ps) if (treat==0 )

regress EASI_w24 treat [pweight=ipw]

local treat_boot=_b[treat]

restore

post ps_1 (`treat_boot')

}

postclose ps_1

bstat using ps_1 , stat(`treat')

mat list e(V)

/*Adjusted via IPTW using the estimated propensity score for continuous IgEstrat */

cap drop P1 P2 P3 P4 int_1 int_2 int_3 int_4

cap drop ps ipw

logit treat agestrat IgEbaseline1 EASI_1 if EASI_w24!=.

predict ps

gen ipw=1/ps

replace ipw=1/(1-ps) if (treat==0 )

regress EASI_w24 treat [pweight=ipw]

*Mu_1:

gen P1=(EASI_w24*treat)/ps

gen P2=treat/ps

summ P1

local C1 = r(sum)

summ P2

local C2 = r(sum)

local C2=`C2'^(-1)

scalar mu_1=`C1'*`C2'

di mu_1

*Mu_0:

gen P3=(EASI_w24*(1-treat))/(1-ps)

gen P4=(1-treat)/(1-ps)

summ P3

local C3 = r(sum)

summ P4

local C4 = r(sum)

local C4=`C4'^(-1)

scalar mu_0=`C3'*`C4'

di mu_0

di mu_0-mu_1

gen int_1=treat/ps

summ int_1

scalar w1 = (1/60)*r(sum)

gen int_2=(1-treat)/(1-ps)

summ int_2

scalar w0 = (1/60)*r(sum)

scalar K01 = 1

scalar K11 = 1

gen int_3=(((EASI_w24-mu_1)^2)*treat)/(ps*ps)

summ int_3

scalar int_3_sum=r(sum)

gen int_4=(((EASI_w24-mu_0)^2)*(1-treat))/((1-ps)*(1-ps))

summ int_4

scalar int_4_sum=r(sum)

scalar V_un = (((K11*K11)/(w1*w1))*(1/60)*int_3_sum)+((((K01*K01)/(w0*w0)))*(1/60)*int_4_sum)

di V_un

forvalues i=1/60{

local cov1 = EASI_1 in `i'

local cov2 = IgEbaseline1 in `i'

local cov3 = agestrat in `i'

mat define x`i' = [1,`cov1' , `cov2', `cov3']'

local mult`i' = ((EASI_w24-mu_1)*treat*(1-ps))/ps in `i'

mat define x1_`i'=x`i'*`mult`i''

}

mat define x1_sum = x1_1

mat list x1_sum

forvalues i=2/60{

mat define x1_sum= x1_sum+x1_`i'

}

forvalues i=1/60{

local cov1 = EASI_1 in `i'

local cov2 = IgEbaseline1 in `i'

local cov3 = agestrat in `i'

mat define x`i' = [1,`cov1' , `cov2', `cov3']'

local Tmult`i' = ((EASI_w24-mu_0)*(1-treat)*(ps))/(1-ps) in `i'

mat define x2_`i'=x`i'*`Tmult`i''

}

mat define x2_sum = x2_1

forvalues i=2/60{

mat define x2_sum= x2_sum+x2_`i'

}

mat define v_hat = ((((K11/w1)*(1/60)))*x1_sum) + ((((K01)/w0*(1/60)))*x2_sum)

mat list v_hat

forvalues i=1/60{

local cov1 = EASI_1 in `i'

local cov2 = IgEbaseline1 in `i'

local cov3 = agestrat in `i'

mat define x`i' = [1,`cov1' , `cov2', `cov3']'

local Mult_`i' = (ps)*(1-ps) in `i'

mat define x_x_`i'=x`i'*x`i''

mat define ext_`i' = x_x_`i'*`Mult_`i''

}

mat define M1_sum = ext_1

forvalues i=2/60{

mat define M1_sum= M1_sum+ext_`i'

}

mat define M1_int = ((1/60)*M1_sum)

mat define M1=inv(M1_int)

forvalues i=1/60{

local cov1 = EASI_1 in `i'

local cov2 = IgEbaseline1 in `i'

local cov3 = agestrat in `i'

mat define x`i' = [1,`cov1' , `cov2', `cov3']'

local Mult_`i' = (treat -ps)^2 in `i'

mat define x_x_`i'=x`i'*x`i''

mat define ext_`i' = x_x_`i'*`Mult_`i''

}

mat define M2_sum = ext_1

forvalues i=2/60{

mat define M2_sum= M2_sum+ext_`i'

}

mat define M2 = M1*((1/60)*M2_sum)*M1

mat define nvar_delta = V_un - (v_hat'*((2*M1)-M2)*v_hat)

mat list nvar_delta

di nvar_delta[1,1]/60

di sqrt(nvar_delta[1,1]/60)

*CI using normal distribution:

di -6.579443+1.96* 3.1896272

di -6.579443-1.96* 3.1896272

local t= -6.579443/3.1896272

di 2*(1-normal(abs(`t')))

drop ps

drop ipw

local treat=mu_0-mu_1

postfile ps_2 Treat using ps_2, replace

forvalues i=1(1)10000{

preserve

bsample

logit treat agestrat IgEbaseline1 EASI_1 if EASI_w24!=.

predict ps

gen ipw=1/ps

replace ipw=1/(1-ps) if (treat==0 )

regress EASI_w24 treat [pweight=ipw]

local treat_boot=_b[treat]

restore

post ps_2 (`treat_boot')

}

postclose ps_2

bstat using ps_2 , stat(`treat')

mat list e(V)

/*Sensitivity analysis excluding IgE via adjusted regression*/

regress EASI_w24 treat agestrat EASI_1

local treat = _b[treat]

postfile ad_3 Treat using ad_3, replace

forvalues i=1(1)10000{

preserve

bsample

regress EASI_w24 treat agestrat EASI_1

local treat_boot=_b[treat]

restore

post ad_3 (`treat_boot')

}

postclose ad_3

bstat using ad_3 , stat(`treat')

mat list e(V)

/*Adjusted via IPTW using the estimated propensity score without IgE */

cap drop P1 P2 P3 P4 int_1 int_2 int_3 int_4

cap drop ps ipw

logit treat agestrat EASI_1 if EASI_w24!=.

predict ps

gen ipw=1/ps

replace ipw=1/(1-ps) if (treat==0 )

regress EASI_w24 treat [pweight=ipw]

*Mu_1:

gen P1=(EASI_w24*treat)/ps

gen P2=treat/ps

summ P1

local C1 = r(sum)

summ P2

local C2 = r(sum)

local C2=`C2'^(-1)

scalar mu_1=`C1'*`C2'

di mu_1

*Mu_0:

gen P3=(EASI_w24*(1-treat))/(1-ps)

gen P4=(1-treat)/(1-ps)

summ P3

local C3 = r(sum)

summ P4

local C4 = r(sum)

local C4=`C4'^(-1)

scalar mu_0=`C3'*`C4'

di mu_0

di mu_0-mu_1

gen int_1=treat/ps

summ int_1

scalar w1 = (1/60)*r(sum)

gen int_2=(1-treat)/(1-ps)

summ int_2

scalar w0 = (1/60)*r(sum)

scalar K01 = 1

scalar K11 = 1

gen int_3=(((EASI_w24-mu_1)^2)*treat)/(ps*ps)

summ int_3

scalar int_3_sum=r(sum)

gen int_4=(((EASI_w24-mu_0)^2)*(1-treat))/((1-ps)*(1-ps))

summ int_4

scalar int_4_sum=r(sum)

scalar V_un = (((K11*K11)/(w1*w1))*(1/60)*int_3_sum)+((((K01*K01)/(w0*w0)))*(1/60)*int_4_sum)

di V_un

forvalues i=1/60{

local cov1 = EASI_1 in `i'

local cov2 = agestrat in `i'

mat define x`i' = [1,`cov1' , `cov2']'

local mult`i' = ((EASI_w24-mu_1)*treat*(1-ps))/ps in `i'

mat define x1_`i'=x`i'*`mult`i''

}

mat define x1_sum = x1_1

mat list x1_sum

forvalues i=2/60{

mat define x1_sum= x1_sum+x1_`i'

}

forvalues i=1/60{

local cov1 = EASI_1 in `i'

local cov2 = agestrat in `i'

mat define x`i' = [1,`cov1' , `cov2']'

local Tmult`i' = ((EASI_w24-mu_0)*(1-treat)*(ps))/(1-ps) in `i'

mat define x2_`i'=x`i'*`Tmult`i''

}

mat define x2_sum = x2_1

forvalues i=2/60{

mat define x2_sum= x2_sum+x2_`i'

}

mat define v_hat = ((((K11/w1)*(1/60)))*x1_sum) + ((((K01)/w0*(1/60)))*x2_sum)

mat list v_hat

forvalues i=1/60{

local cov1 = EASI_1 in `i'

local cov2 = agestrat in `i'

mat define x`i' = [1,`cov1' , `cov2']'

local Mult_`i' = (ps)*(1-ps) in `i'

mat define x_x_`i'=x`i'*x`i''

mat define ext_`i' = x_x_`i'*`Mult_`i''

}

mat define M1_sum = ext_1

forvalues i=2/60{

mat define M1_sum= M1_sum+ext_`i'

}

mat define M1_int = ((1/60)*M1_sum)

mat define M1=inv(M1_int)

forvalues i=1/60{

local cov1 = EASI_1 in `i'

local cov2 = agestrat in `i'

mat define x`i' = [1,`cov1' , `cov2']'

local Mult_`i' = (treat -ps)^2 in `i'

mat define x_x_`i'=x`i'*x`i''

mat define ext_`i' = x_x_`i'*`Mult_`i''

}

mat define M2_sum = ext_1

forvalues i=2/60{

mat define M2_sum= M2_sum+ext_`i'

}

mat define M2 = M1*((1/60)*M2_sum)*M1

mat define nvar_delta = V_un - (v_hat'*((2*M1)-M2)*v_hat)

mat list nvar_delta

di nvar_delta[1,1]/60

di sqrt(nvar_delta[1,1]/60)

*CI using normal distribution:

di -6.3948058+1.96* 3.1497406

di -6.3948058-1.96* 3.1497406

local t= -6.3948058/3.1497406

di 2*(1-normal(abs(`t')))

cap drop ps

cap drop ipw

local treat=mu_0-mu_1

postfile ps_3 Treat using ps_3, replace

forvalues i=1(1)10000{

preserve

bsample

logit treat agestrat EASI_1 if EASI_w24!=.

predict ps

gen ipw=1/ps

replace ipw=1/(1-ps) if (treat==0 )

regress EASI_w24 treat [pweight=ipw]

local treat_boot=_b[treat]

restore

post ps_3 (`treat_boot')

}

postclose ps_3

bstat using ps_3 , stat(`treat')

mat list e(V)
